# Mutation profiling of 19 candidate genes in acute myeloid leukemia suggests significance of *DNMT3A* mutations

**DOI:** 10.18632/oncotarget.10240

**Published:** 2016-06-23

**Authors:** Sang-Yong Shin, Seung-Tae Lee, Hee-Jin Kim, Eun Hae Cho, Jong-Won Kim, Silvia Park, Chul Won Jung, Sun-Hee Kim

**Affiliations:** ^1^ Department of Laboratory Medicine, Center for Diagnostic Oncology, Hospital and Research Institute, National Cancer Center, Goyang, Korea; ^2^ Department of Laboratory Medicine, Yonsei University College of Medicine, Seoul, Korea; ^3^ Department of Laboratory Medicine & Genetics, Samsung Medical Center, Sungkyunkwan University School of Medicine, Seoul, Korea; ^4^ Division of Hematology-Oncology, Department of Medicine, Samsung Medical Center, Sungkyunkwan University School of Medicine, Seoul, Korea; ^5^ Green Cross Genome, Yongin, Korea

**Keywords:** acute myeloid leukemia, mutation, next generation sequencing, DNMT3A

## Abstract

We selected 19 significantly-mutated genes in AMLs, including *FLT3*, *DNMT3A, NPM1, TET2, RUNX1, CEBPA, WT1, IDH1, IDH2, NRAS, ASXL1, SETD2, PTPN11, TP53, KIT, JAK2, KRAS, BRAF* and *CBL*, and performed massively parallel sequencing for 114 patients with acute myeloid leukemias, mainly including those with normal karyotypes (CN-AML). More than 80% of patients had at least one mutation in the genes tested. *DNMT3A* mutation was significantly associated with adverse outcome in addition to conventional risk stratification such as the European LeukemiaNet (ELN) classification. We observed clinical usefulness of mutation testing on multiple target genes and the association with disease subgroups, clinical features and prognosis in AMLs.

## INTRODUCTION

Acute myeloid leukemia (AML) is a biologically heterogeneous disease and its clinical behaviors largely vary among different cases. Prognostic stratification in AML is important to determine treatment strategy. A well-proven prognostic factor is cytogenetic abnormality, according to which patients can be categorized as favorable, intermediate and unfavorable cytogenetic groups [1, 2]. Those with inv(16)/t(16;16), t(8;21) and t(15;17) are classified into favorable cytogenetic risk group while those with complex karyotype, t(6;9), inv(3)/t(3;3), −5/del(5q) and −7/del(7q) are classified into adverse cytogenetic risk group. Cases with intermediate cytogenetic risk group are predominantly with normal cytogenetics (CN-AMLs), constituting about 50% of all AML cases.

Recently, mutations in *FLT3, NPM1* and *CEBPA* have been shown to have significant prognostic impact were suggested to be included in the risk stratification system by European LeukemiaNet (ELN) [2]. In addition, several studies have suggested prognostic implication of mutations in other genes. *DNMT3A* mutations (about one third of intermediate cytogenetic risk group) were suggested to be associated with adverse outcomes among intermediate-risk cytogenetic group [3]. *IDH1/2,* which encodes the enzyme isocitrate dehydrogenase, was recently shown to be mutated in about 16% of all AMLs and have significant prognostic impact in CN-AML with mutated *NPM1* without *FLT3*-ITD [4]. In some studies, *TET2* mutation was suggested to be associated with poor prognostic impact in CN-AML or intermediate cytogenetic risk group [5, 6]. *KIT* mutation was suggested to have adverse prognostic impact in AML with t(8;21) or inv(16)/t(16;16) [7]. *WT1* mutation in AML was found about 10-15% and associated with adverse prognostic impact in some studies [8, 9]. *TP53* mutation is frequently associated with therapy-related myeloid neoplasm with adverse prognostic impact [10, 11]. *RUNX1* was found ~20% of *de novo* CN-AML with short overall survival and relapse free survival [11]. *ASXL1, SETD2, JAK2, KRAS, PTPN11, NRAS, BRAF* and *CBL* were found in AML and other myeloproliferative neoplasms such as myelodysplastic syndrome/myeloproliferative neoplasm (MDS/MPN) with varying frequency and prognostic impact [12–14].

In the present study, we have comprehensively reviewed recent literatures on AML mutations and selected target genes which show relatively high frequencies (*NPM1, FLT3, IDH1/2, CEBPA, DNMT3A, WT1*), adverse prognostic impact (*TET2, ASXL1, NRAS, KRAS, KIT)* and associations with secondary/therapy related myeloid neoplasm/cytogenetic abnormalities (*JAK2, TP53, PTPN11, BRAF, CBL, SETD2*) [5, 12, 14–22]. And we investigated mutation profiles of these 19 genes selected genes [5, 12, 14–22] and their association with clinical parameters and outcomes in Korean patients with AML. Our data suggested a significant impact of *DNMT3A* mutations over other conventional prognostic factors. Investigation on a relatively homogeneous population from a single institute will provide valuable information to the current knowledge on the impact of gene mutations on AMLs, together with molecular epidemiological insights on this regional population.

## RESULTS

### Clinical characteristics of 114 AML cases

Demographics and clinical characteristics of enrolled cases are summarized in Table 1 and Supplementary Table S1. Seventy male and 44 female were enrolled. Ninety-eight adult patients (>19 years) and 16 childhood (<20 years) AML patients were enrolled. Ninety-three cases were *de novo* AMLs and 12 cases were secondary or relapsed AMLs. Two cases were secondary AMLs evolved from primary myelofibrosis and polycythemia vera.

**Table 1:** Clinical and cytogenetic characteristics of enrolled cases

	CN-AML (n=77)	CA-AML (n=26)	Unknown* (n=11)	Total
**Male/Female**	48/29	16/10	6/5	70/44
**Age, years (mean±SD)**	51.4±19.3	40.8±25.6	61.7±15.5	50.0±21.2
**Complete remission**	60/63(95.2%)	20/21(95.2%)	2/2(100%)	82/86(95.3%)
**Relapse**	27/60(45.0%)	9/20(45.0%)	1/2(50%)	37/82(45.1%)
**Bone marrow transplantation**	35/77(45.5%)	14/26(53.8%)	2/2(100%)	51/105(48.6%)
**Age group**				
1-19 years	7	9		16
20-39 years	10	1	1	12
40-59 years	29	8	3	40
≥60	31	8	7	46
**Secondary or relapse**	4	8		12
***De novo***	73	18	2	93
**2008 WHO classification**				
**AML with t(8;21)(q22;q22)**		4		4
**AML with inv(16)(p13q22)**		2		2
**AML with t(15;17)(q22;q21)**		2†		2
**AML with myelodysplasia-related changes**	30	13		43
**AML, not otherwise specified**				
AML with minimal differentiation(M0)	4	1		5
AML without maturation(M1)	8	2		10
AML with maturation(M2)	20	0		20
Acute myelomonocytic leukemia(M4)	9	1		10
Acute monoblastic/monocytic leukemia(M5)	2	1		3
Acute erythroid leukemia(M6)	2			2
Acute megakaryoblastic leukemia(M7)	2	0		2
Unknown*			11	11
**Cytogenetic risk group**				
Favorable	0	8		8
Intermediate	77	7		84
Unfavorable		11		11
Unknown*			11	11

* Cytogenetic results were unavailable or inconclusive

† One patient had normal karyotype but fluorescence in situ hybridization and reverse transcript PCR for *PML-RARA* rearrangement were positive

### Categorization and cytogenetic risk group

Of the 114 patients enrolled, 11 had missing or inconclusive cytogenetic results. Among the 103 cases with unequivocal cytogenetic information, 77 were CN-AMLs and 26 were cytogenetically abnormal AMLs (CA-AMLs). As to recurrent cytogenetics, four cases with t(8;21)(q22;q22), 2 cases with inv(16)(p13q22) and 2 cases with t(15;17)(q22;q21) were also enrolled.

When categorized by the 2008 WHO classification of hematologic malignancies [23], AML-MRC was the most common type (43/114, 37.7 %). AML with maturation (20/52), AML without maturation (10/52) and acute myelomonocytic leukemia (10/52) were most common in AML-NOS.

According to the ELN prognostic stratification [2], eight patients were categorized into favorable, 84 patients were into intermediate, and 11 patients were into unfavorable cytogenetic risk groups. Among the 43 AML-MRC cases, 13 cases had cytogenetic abnormalities. Among these, 12 cases including those with −5/del(5q), −7/del(7q) and complex cytogenetic abnormalities were categorized as poor cytogenetic risk group according to the ELN prognostic stratification.

### Information on treatment

Information on treatment regimen was available in 89 adult AML cases. A total of 74 patients received the intensive chemotherapy (high dose cytarabine and idarubicin). Nine patients had received low intensity chemotherapy (hydroxyurea, decitabine, azacitidine etc.), and six patients did not receive chemotherapy due to early death or unstable clinical status. Among 43 AML-MRC patients, 28 (73.6%) had received intensive chemotherapy, and 5 (13.2%) received low intensity chemotherapy. Five AML-MRC patients (13.2%) did not receive chemotherapy.

Among 56 intermediated risk group (i.e. *de novo* CN-AML with other than mutated *NPM1* without *FLT3*-ITD) patients, 46 (81.2%) had received intensive chemotherapy, and 6 (10.7%) received low intensity chemotherapy. Four AML-MRC patients (7.1%) did not receive chemotherapy.

### Mutation spectra, hotspots and co-occurrence

The overall fold coverage (read length/number of reads) was over 1000× (range: 1030-4830×) in all target regions except for some focal regions in *KRAS, CEBPA* and *TET2* (Supplementary Figure S1). Among the 199 variations called and filtered, 166 (83.4%) were confirmed by Sanger sequencing (Supplementary Table S2 and S3). Because 3′-part of *CEBPA* was sequenced with very low coverage, Sanger sequencing results were integrated as a supplementation. The detection rate of *FLT3*-ITD by massively parallel sequencing was low (9 of 34 positive cases), especially in those with low mutant allele proportion, so clinical testing results were also integrated.

**Figure 1:**
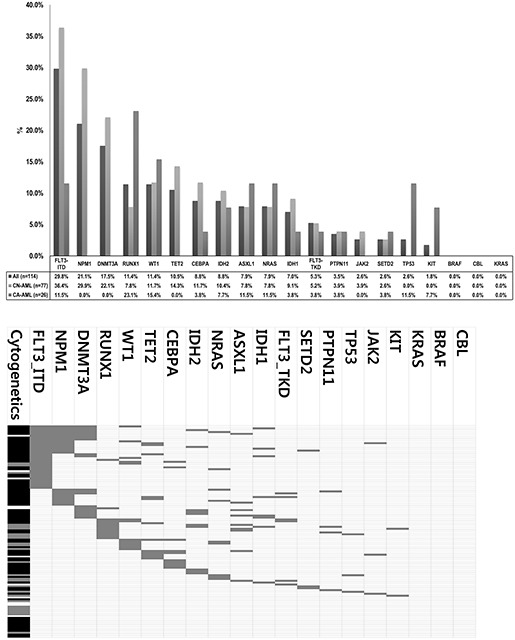
Mutational profiles of AML cases analyzed **A.** Mutation frequencies according to chromosome abnormalities. **B.** Mutation status in individual cases and co-occurrence among other mutations and chromosome abnormalities. Gray-color box indicates mutated case. For cytogenetic abnormalities, Black-color box indicates CN-AML, Gray-color box indicates CA-AML, White-color box indicates other AML (i.e. unknown cytogenetics).

**Figure 2:**
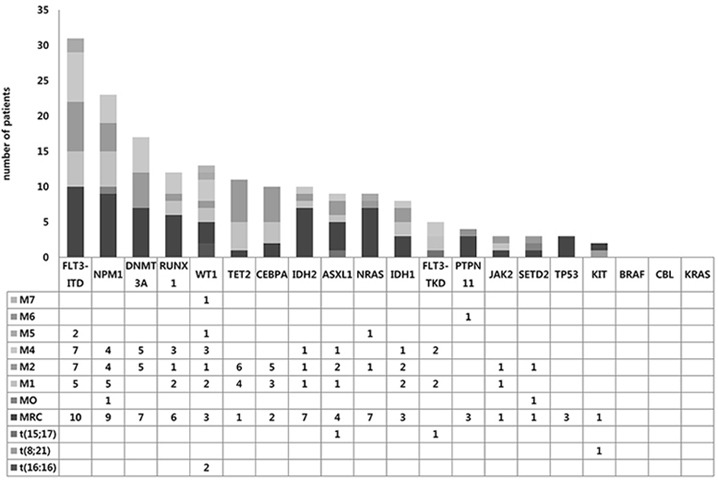
Mutation spectrums according to the 2008 WHO Classification Proportion of mutations in each gene according to AML subgroups.

As a total, ninety-two (80.7%) cases had at least one mutation in the 19 genes investigated, suggesting that this panel could be used effectively as initial workup of AMLs. For all confirmed mutations, we statistically analyzed mutation profiles according to clinical and cytogenetic subgroups (Figure 1 and 2).

In CN-AML, *FLT3*-ITD was the most common mutation (36.4%), followed by *NPM1* and *DNMT3A* mutations (29.9% and 22.1%, respectively). As previously reported, *NPM1* mutations were found only in CN-AML and the majority (19/24, 79.2%) was type A (960insTCTG) mutation, while three Type B (960insCATG) and two Type D (960insCCTG) mutations were identified. *DNMT3A* mutations were found only in CN-AML, with R882H mutation being most common (9/20, 45%) as previously reported and other mutations being scattered across proline-tryptophan rich, zinc finger and methytransferase domains (Supplementary Figure S2). *RUNX1* mutations were found in 13 cases (15 variation) with frameshift being the most predominant mutation type (10/15, 66.6%) (Supplementary Figure S3). *WT1* mutations were detected in 13 cases (11.4%) overall, especially in codons 381 and 382 on exon 7 (Supplementary Figure S4). *TET2* mutations were distributed throughout coding regions, and loss-of-function mutations including nonsense (9/17) and frameshift (5/17) were commonly observed (Supplementary Figure S5). All *IDH1* mutations detected were R132C (100%, 9/9), and *IDH2* mutations were detected either as R140Q (60%, 6/10) or R172K (30%, 3/10). *NRAS* mutations were detected in 10 cases (7.9%) overall and all were missense mutations in codons 12, 13 and 61 (Supplementary Figure S6). *ASXL*1 mutations were clustered around 5′-part of exon 12 (Supplementary Figure S7). *PTPN11* mutations were found in 4 cases (3.5%) overall, with a predominant occurrence in exon 3 (Supplementary Figure S8). *KIT* mutations were detected only in two cases; AML with t(8;21) and AML-MRC. *TP53* mutations were detected in three cases with AML-MRC.*JAK2* V617F mutation was detected in secondary AMLs evolved from polycythemia vera and primary myelofibrosis, while one *de novo* AML (AML without maturation) case also harbored *JAK2* V617F mutation. *SETD2* mutations were detected in three (2.6%) *de novo* AMLs. Only one case with double *CEBPA* mutations was found in this study, whereas 9 cases had only a single *CEBPA* mutation. No mutation was detected in the *CBL, KRAS* and *BRAF* genes.

*NPM1* mutations tended to co-occur with *DNMT3A* (*P*<0.001) and *FLT3*-ITD mutations (*P*<0.001) while *DNMT3A* mutation was frequently concurrent with *IDH1/2* mutation (*P*=0.002*)* (Table 2). In line with several previous reports [24–28], high degrees of mutual exclusivity existed between *IDH1* and *IDH2, WT1* and *IDH1*/*2*, and *DNMT3A* and *CEBPA* mutations. We also newly observed a high exclusivity between *DNMT3A* and *RUNX1* mutations.

**Table 2:** Co-occurrence of gene mutations

	***NPM1***					***NPM1***			
***DNMT3A***		**wild**	**mutant**	***P***	***FLT3*-ITD**		**wild**	**mutant**	***P***
	**wild**	81	13	*<.001*		**wild**	71	9	*<.001*
	**mutant**	9	11			**mutant**	19	15	
	***FLT3*-ITD**					***WT1***			
***DNMT3A***		**wild**	**mutant**	***P***	***IDH1/2***		**wild**	**mutant**	***P***
	**wild**	70	24	*.003*		**wild**	84	13	*.211*
	**mutant**	10	10			**mutant**	17	0	
	***CEBPA***					***IDH1/2***			
***DNMT3A***		**wild**	**mutant**	***P***	***TET2***		**wild**	**mutant**	***P***
	**wild**	84	10	*.204*		**wild**	86	16	*0.690*
	**mutant**	21	0			**mutant**	11	1	
	***IDH1/2***					***RUNX1***			
***DNMT3A***		**wild**	**mutant**	***P***	***ASXL1***		**wild**	**mutant**	***P***
	**wild**	85	9	*.002*		**wild**	95	10	*.066*
	**mutant**	12	8			**mutant**	6	3	
	***RUNX1***					***IDH1***			
***DNMT3A***		**wild**	**mutant**	***P***	***IDH2***		**wild**	**mutant**	***P***
	**wild**	82	12	*.459*		**wild**	97	7	*.532*
	**mutant**	19	1			**mutant**	9	1	

### Different mutation frequencies according to AML subgroups

*FLT3*-ITD mutations were frequently found in CN-AMLs than CA-AMLs (36.4% *vs* 11.5%; *P*=0.017) (Table 3). *NPM1* mutations (29.9% *vs.* 0.0%; *P*=0.002) and *DNMT3A* mutations (22.1% *vs.* 0%; *P*=0.006) were exclusively found in CN-AML. *FLT3*-ITD, *NPM1, DNMT3A, IDH1/2, TET2, WT1* and *RUNX1* mutations were relatively common in *de novo* AMLs, while *RUNX1, NRAS*, *PTPN11, JAK2, WT1* and *IDH1/2* mutations were so in secondary or relapsed AMLs (Table 3). Nonetheless, only *JAK2* mutation showed statistical significance.

**Table 3:** Mutation profile according to cytogenetic abnormality and *de novo* and secondary/relapsed cases

Gene	CN-AML (n=77)	CA-AML(n=26)	*P*	*De novo* (n=93)	Secondary or relapsed (n=12)	*P*
***ASXL1***	6(7.8%)	3(11.5 %)	.689	8(8.6%)	1(8.3%)	1.000
***BRAF***	0(0.0%)	0(0.0 %)	-	0(0.0%)	0(0.0%)	-
***CBL***	0(0.0%)	0(0.0 %)		0(0.0%)	0(0.0%)	-
***CEBPA***	9(11.7%)	1(3.8 %)	.445	10(10.8%)	0(0.0%)	.600
***DNMT3A***	17(22.1%)	0(0.0 %)	.006	17(18.3%)	0(0.0%)	.208
***FLT3*-ITD**	28(36.4%)	3(11.5 %)	.017	31(33.3%)	1(8.3%)	.101
***FLT3*-TKD**	4(5.2%)	1(3.8 %)	1.000	5(5.4%)	0(0.0%)	1.000
***IDH1***	7(9.1%)	1(3.8 %)	.676	7(7.5%)	1(8.3%)	1.000
***IDH2***	8(10.4%)	2(7.7 %)	1.000	9(9.7%)	1(8.3%)	1.000
***JAK2***	3(3.9%)	0(0.0 %)	.570	1(1.1%)	2(16.7%)	.034
***KIT***	0(0.0%)	2(7.7 %)	.062	1(1.1%)	1(8.3%)	.216
***KRAS***	0(0.0%)	0(0.0 %)	-	0(0.0%)	0(0.0%)	-
***NPM1***	23(29.9%)	0(0.0 %)	.002	23(24.7%)	0(0.0%)	.064
***NRAS***	6(7.8%)	3(11.5 %)	.689	6(6.5%)	3(25.0%)	.065
***PTPN11***	3(3.9%)	1(3.8 %)	1.000	2(2.2%)	2(16.7%)	.063
***RUNX1***	6(7.8%)	6(23.1 %)	.070	9(9.7%)	3(25.0%)	.138
***SETD2***	2(2.6%)	1(3.8 %)	1.000	3(3.2%)	0(0.0%)	1.000
***TET2***	11(14.3%)	0(0.0 %)	.061	11(11.8%)	1(8.3%)	1.000
***TP53***	0(0.0%)	3(11.5 %)	.015	2(2.2%)	1(8.3%)	.308
***WT1***	9(11.7%)	4(15.4 %)	.734	11(11.8%)	2(16.7%)	.642

Among the cases with no recurrent cytogenetic abnormalities according to the 2008 WHO Classification, 92.3% (48/52) of AML, not otherwise specified (AML-NOS) and 81.4% (35/43) of AML-MRC had at least one mutations in the 19 genes examined (Table 4).*NRAS* mutation was more frequent in AML-MRC (16.3% *vs.* 3.8%, *P*=.074), whereas *TET2* mutation was less frequent in AML-MRC than AML-NOS (2.3% *vs.* 19.2%, *P*=.011). Similar with previous reports [10, 29], we observed a relatively high frequency of *TP53* mutations in AML-MRC (0.0% *vs.* 7.1%, *P*=.089).

**Table 4:** Comparison of mutation profiles and clinical characteristics between AML-MRC and AML-NOS

	AML-NOS(n=52)	AML-MRC(n=43)	*P*
***Age***	46.9±21.2	52.9±20.6	.112
***Sex(male/female)***	33/19	28/15	.867
***Complete remission***	44/46(95.7%)	28/30(94.7%)	.645
***Relapse***	15/44(34.1%)	19/28(57.6%)	.005
***Bone marrow transplantation***	29/52(55.8%)	15/43(34.9%)	.042
***Gene mutation***			
***ASXL1***	4(7.7%)	4(9.3%)	1.000
***CEBPA***	8(15.4%)	2(4.7%)	.107
***DNMT3A***	10(19.2%)	7(16.3%)	.709
***FLT3-*ITD**	21(40.3%)	10(23.3%)	.076
***FLT3-*TKD**	4(7.7%)	0(0.0%)	.124
***IDH1***	5(9.6%)	3(7.0%)	.725
***IDH2***	3(5.8%)	6(16.3%)	.177
***NPM1***	14(26.9%)	9(20.9%)	.497
***NRAS***	2(3.8%)	7(16.3%)	.074
***PTPN11***	1(1.9%)	3(9.3%)	.325
***RUNX1***	6(11.5%)	6(14.0%)	.764
***SETD2***	2(3.8%)	1(2.3%)	1.000
***TET2***	10(19.2%)	1(2.3%)	.011
***TP53***	0(0.0%)	3(7.0%)	.089
***WT1***	8(15.4%)	3(7.0%)	.335
***JAK2***	2(3.8%)	1(2.3%)	1.000

### Complete remission and relapse rates according to mutation status

Complete remission (CR) rate was 95.3% (82/86) and relapse rate was 45.1% (37/82). *RUNX1* mutation was associated with low complete remission rate (57.1% *vs*. 100%, *P=.001*). We observed that *NRAS* and *DNMT3A* mutations were associated with high relapse rates (41.7% *vs*. 100%, *P*=.037 for *NRAS*; 38.9*%, vs.* 80.0%, *P*=.034, for *DNMT3A*) in adult *de novo* AML (Supplementary Table S4).

### Overall survival according to mutation status

We investigated overall survival (OS) according to mutation status in adult (>19 years) *de novo* AMLs with normal karyotype (n=66) or in intermediate cytogenetics risk group (n=68) and found that *DNMT3A* mutation was significantly associated with short OS in a multivariate cox regression model (Supplementary Table S5).

**Figure 3:**
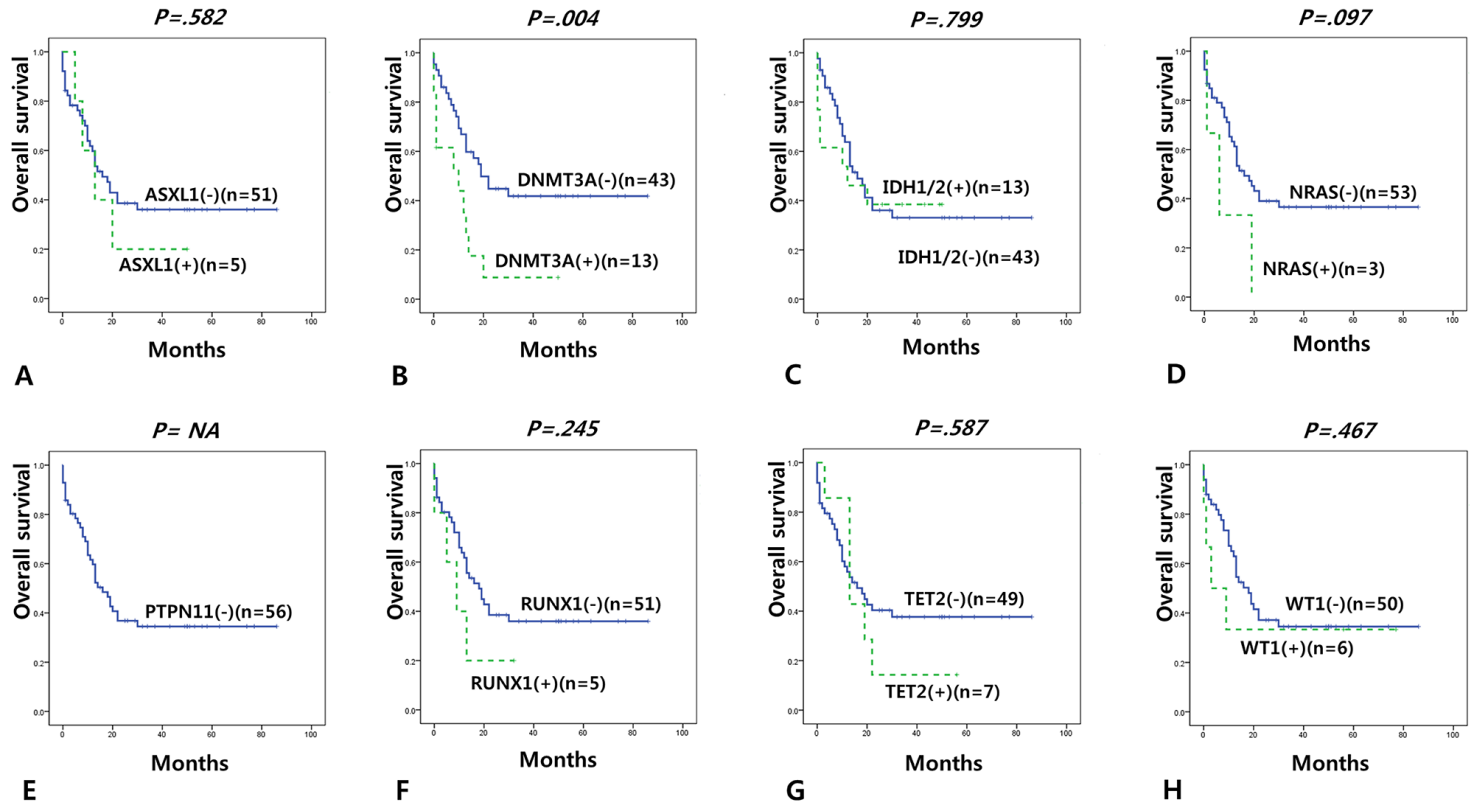
Overall survival according to gene mutations in intermediate group (adult *de novo* CN-AML with other than mutated *NPM1* without *FLT3*-ITD) according to the ELN classification

We further reclassified adult cases according to the ELN classification which incorporates *FLT3*-ITD, *NPM1* and *CEBPA* mutation status. *DNMT3A* and *PTPN11* mutation were associated with short OS in the favorable risk ELN group (i.e. *de novo* CN-AML with mutated *NPM1* without *FLT3*-ITD or *de novo* CN-AML with doubly-mutated *CEBPA*, n=10) (Supplementary Figure S9). *DNMT3A* mutations were associated with short OS in intermediate risk group (i.e. *de novo* CN-AML with other than mutated *NPM1* without *FLT3*-ITD, n=56) (Figure 3).

### Relapse free survival according to mutation status

**Figure 4:**
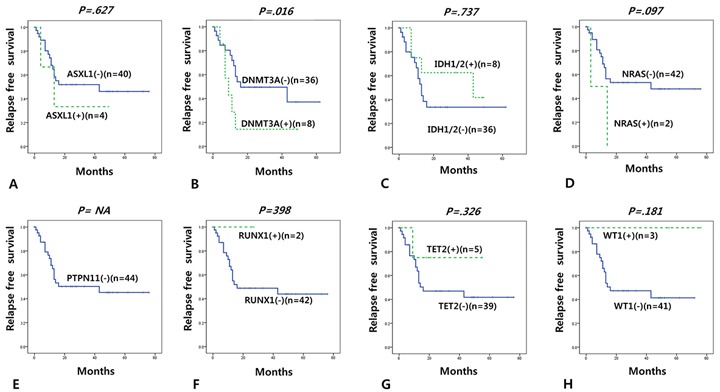
Relapse free survival according to gene mutations in intermediate group (adult *de novo* CN-AML with other than mutated *NPM1* without *FLT3*-ITD) according to the ELN classification

We also investigated relapse free survival (RFS) according to mutation status in 53 adult (>19 years) *de novo* CN-AML cases. In a multivariable cox regression model, only *DNMT3A* mutation was associated with short RFS (Supplementary Table S6). We observed the similar results in analysis of RFS in 55 adult (>19 years) *de novo* intermediate cytogenetic risk group AML cases (Supplementary Table S6). When re-grouped by the ELN classification, *DNMT3A* was associated with short RFS in the intermediate risk ELN group (Figure 4 and Supplementary Figure S10).

### Clinical outcomes of adult de novo AML-MRC and AML-NOS

AML-MRC had significantly shorter RFS than AML-NOS, while OS was not significantly different (Supplementary Figure S11).

## DISCUSSION

Considering the high prevalence of gene mutations in AML their prognostic impact and disease monitoring, mutation profiling at initial diagnosis may be necessary in addition to the conventional workups [4, 6, 30]. Recent high throughput sequencing technologies may be useful for detecting mutations in a number of target genes [19, 31, 32]. Compared to the whole genome or exome sequencing with highly sophiscated, time-consuming and intensive efforts, targeted gene sequencing may be a practical and ideal method for clinical testing in AML, with low cost, short turnaround time and less burden for bioinformatics [31, 33, 34]. Through a custom targeted panel, we could detect most of the target sites with high fold coverages (>1,000) and more than 80% of cases had at least one mutation in the genes investigated, suggesting the targeted sequencing strategy could be useful for initial workup. However, we had suboptimal coverages for *CEBPA*, where guanine-cytosine (GC) content is high and PCR amplification is problematic, and *FLT3*-ITD region, where large tandem duplication is common and short amplicon sequencing can miss some mutations. So for such mutations, conventional Sanger sequencing or PCR-fragment length analysis may be needed as a supplement [35].

As a whole, we observed mutation frequencies similar to previous studies in Western countries [3, 6, 10, 25, 27, 30] as well as in Asian countries. Wang *et al* [36] reported high mutation rates (>10%) in *CEBPA, NPM1, DNMT3A, FLT3-ITD, NRAS, IDH2* and *WT1*. Kihara *et al* [37] reported high mutation rates of *FLT3, NPM1, CEBPA, DNMT3A* and *KIT* mutations in Japanese patients with AML. Some studies focusing on a single or a few genes have been reported in Korean patients with AML. *DNMT3A* R882 was detected 7.5% [38] and 18.3% [39] in Korean patients with CN-AML. Ahn *et al* reported that mutation rates for *IDH1/2, TET2, NRAS* and *WT1* were 16.5%, 8.7%, 6.1% and 14.8%, respectively, in 115 Korean patients with CN-AML [39].

The impact of *FLT3*-ITD, *NPM1* and *CEBPA* mutations on clinical outcome are well-established now, as implemented in the ELN classification [2, 28]. However, we observed particularly significant impact of *DNMT3A* mutations in the favorable or intermediate risk ELN group. As well, relapse rate of *DNMT3A*-mutated cases was significantly higher than unmutated cases (80.0% *vs*. 38.9%, *P=0.034*). Ahn *et al* [39] also reported poor prognostic impact (short OS, short RFS and high relapse rate) of *DNMT3A* R882 mutations in Korean patients with CN-AML after hematopoietic cell transplantation. Other previous studies also support that *DNMT3A* mutation can be an important prognostic factor [25, 26, 40–42] although some studies failed to find such associations [38, 43]. *DNMT3A* mutation was known to be more frequent in CN-AMLs, and we surprisingly observed a high exclusivity of the mutations in CN-AMLs compared CA-AMLs (22.1% *vs.* 0%). This might be due to the small number of CA-AMLs analyzed but should be further investigated in an extended set of patients. Considering the high positive rates and possible clinical implications, we may suggest that *DNMT3A* can be used in routine diagnostics and as an additional factor to the ELN risk stratification. We also observed possible prognostic implication of some genes with lower frequencies, including *WT1, PTPN11, NRAS* and *ASXL1*. Comprehensive mutation profiling may be needed when clinical implication of these rare mutations is established by future large studies and meta-analyses.(35)

In conclusion, we observed the recurrent mutation profile and their clinical significance using Korean AML patients. Although relatively small number of enrolled cases and heterogeneous population, it is first comprehensive mutation analysis using Korean patients. Considering the wide heterogeneity in mutational spectrums and clinical characteristics, it will be essentially needed to accumulate more data on the mutational profile and clinical associations in AMLs from different centers and working groups.

## MATERIALS AND METHODS

### Study population

We obtained bone marrow samples from 114 patients with AML diagnosed at Samsung Medical Center from 2008 to 2012, as well as peripheral blood samples from 4 healthy controls. Genomic DNA was isolated using the Wizard Genomic DNA Purification kit (Promega, Madison, WI, USA). Diagnosis are based on 2008 WHO classification of tumors of haematopoietic and lymphoid tissues [23]. AML-MRC was defined as AML with morphological features of myelodysplasia or a prior history of a myelodysplastic syndrome (MDS) or myelodysplastic/myeloproliferative neoplasm (MDS.MPN), or MDS-related cytogenetic abnormalities, and absence of the specific genetic abnormalities. AML-NOS were classified according to morphology, cytochemical statin and/or immunophenotypic results. Chromosome study was performed with a standard protocol and karyotypes were described according to the International System for Human Cytogenetic Nomenclature (ISCN) 2009. The Institional Review Board at Samsung Medical Center approved the current study (IRB No. 2014-05-016-002).

### Targeted high-throughput sequencing

From a literature review, we selected 19 genes significantly mutated in AML [5, 12, 15, 16]]; they included *FLT3*-ITD*, FLT3*-TKD, *DNMT3A, NPM1, TET2, RUNX1, CEBPA, WT1, IDH1, IDH2, NRAS, ASXL1, SETD2, PTPN11, TP53, KIT, JAK2, KRAS, BRAF* and *CBL* (Supplementary Table S7). Custom target enrichment and amplicon library preparation was done using the Access Array System (Fluidigm, South San Francisco, CA, USA),[18] followed by massively parallel sequencing on the Miseq System (Illumina, San Diego, CA, USA) [44].

### Analysis of high-throughput sequencing data

A schematic overview of bioinformatics analysis is presented in Supplementary Figure S12. After quality control by the SAMtool and fastQC softwares,(46) raw files were aligned to the human reference genome (build GRCh37) using the Burrows-Wheeler Aligner (BWA) with default parameters.(47) Local alignment was done with the Genome Analysis Toolkit (GATK), followed by variant calling by the GATK-Haplotype caller and Varscan algorithms.(48) Read depth and coverage were estimated using the BEDTools.(49) Called variants were annoatated using the SnpEff software(50) and filtered according to the following strategy; i) variation calls detected in normal healthy control were excluded, ii) only missense, nonsense, framshift, start gain and splice site mutations were included, iii) variations found over 0.1% of general population in public databases (NCBI dbSNP and ESP6000)(51) were excluded, iv) only variations with allele frequecy >20% in GATK-Haplotype caller and/or Varscan were included, and v) variants were further filtered by visual inspection on Inegrative Genomics Viwer (IGV) [45]. SIFT and PolyPhen softwares [46, 47] were used for *in*-*silico* analysis of damaging effects of missense variations.

### Crosscheck and validation by Sanger sequencing

Variants filtered by the strategy mentioned above were further confirmed by Sanger sequecing. For *FLT3-*ITD, *FLT3-*TKD*, JAK2* V617F*, KIT, CEBPA* and *NPM1* mutations, we integrated clinical testing results wherever available; all these were tested by Sanger sequencing and *FLT3*-ITD was double-checked by PCR and fluorescent fragment length analysis. Primers used for Sanger sequecing are summarized in Supplementary Table S8. Sequencing was performed using the BigDye Terminator Cycle Sequencing Ready Reaction Kit on an ABI Prism 3730 Genetic Analyzer (Applied Biosystems, Foster City, CA, USA). The sequence data was analyzed using the Sequencher software (Gene Codes Corp., Ann Arbor, MI, USA).

### Statistical analysis and clinical parameters

Statistical analysis was performed using the PASW Statistics 20.0 software (IBM, Armonk, NY, USA). Significance between categorical data was calculated by Chi-square or Fisher's exact tests. Differences in survival between mutation groups were analyzed by Kaplan–Meier estimates. Multivariable Cox regression analysis was performed to examine the impact of mutations along with other clinical variables. OS was measured from the time from diagnosis to death or last follow-up. RFS was defined only for patients achieving CR, measured from the date of achievement of a remission until the date of relapse or death from CR, death or any cause. A *P*-value of <0.05 was considered as statistically significant.

## SUPPLEMENTARY FIGURES AND TABLES








