# Supplementary material for: Mutation profiling of 19 candidate genes in acute myeloid leukemia suggests significance of DNMT3A mutations
Source: Oncotarget. 2016 Jun 23;7(34):54825–37. doi: 10.18632/oncotarget.10240 (PMC5342384; doi:10.18632/oncotarget.10240)
Supplement: Supplementary file 1 [file oncotarget-07-54825-s001.pdf]

Mutation profiling of 19 candidate genes in acute myeloid leukemia suggests significance of *DNMT3A* mutations

SUPPLEMENTARY FIGURES AND TABLES

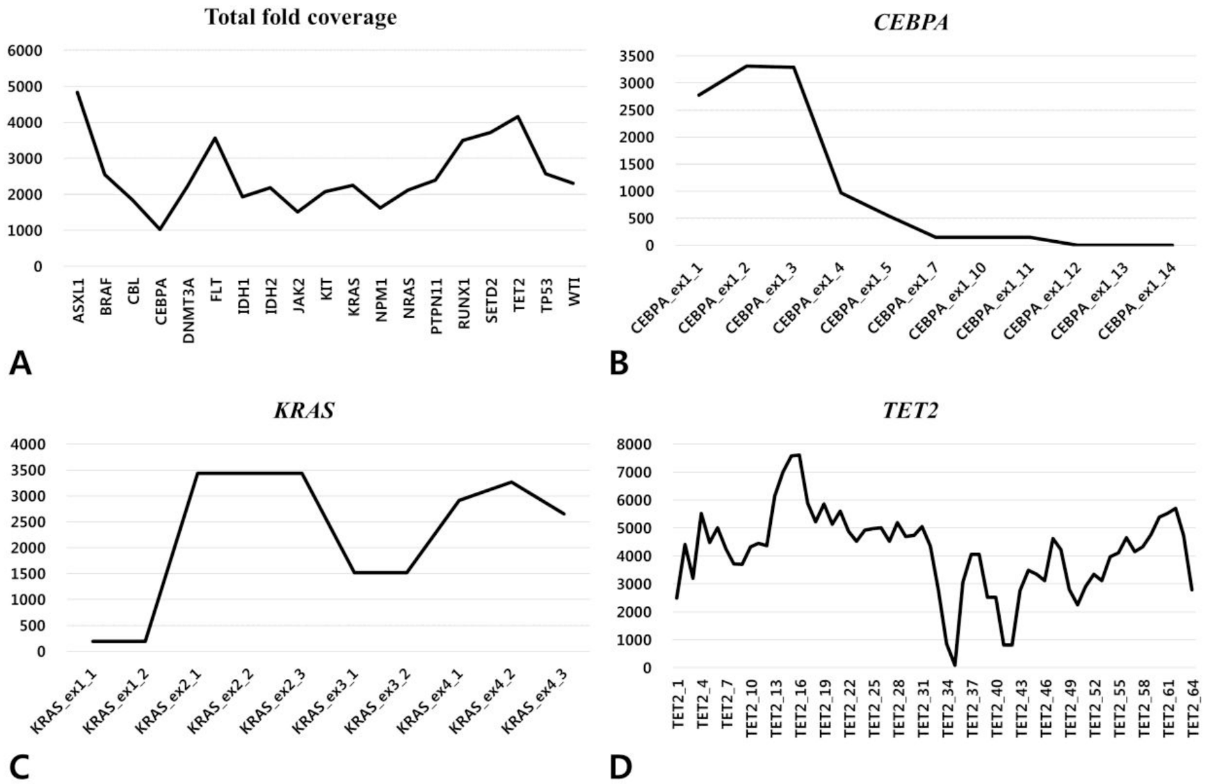

Supplementary Figure S1: Distribution of fold coverage. **A.** Average coverage all across genes. **B.** Coverage of individual amplicons on *CEBPA*. **C.** Coverage of individual amplicons on *KRAS*. **D.** Coverage of individual amplicons on *TET2*.

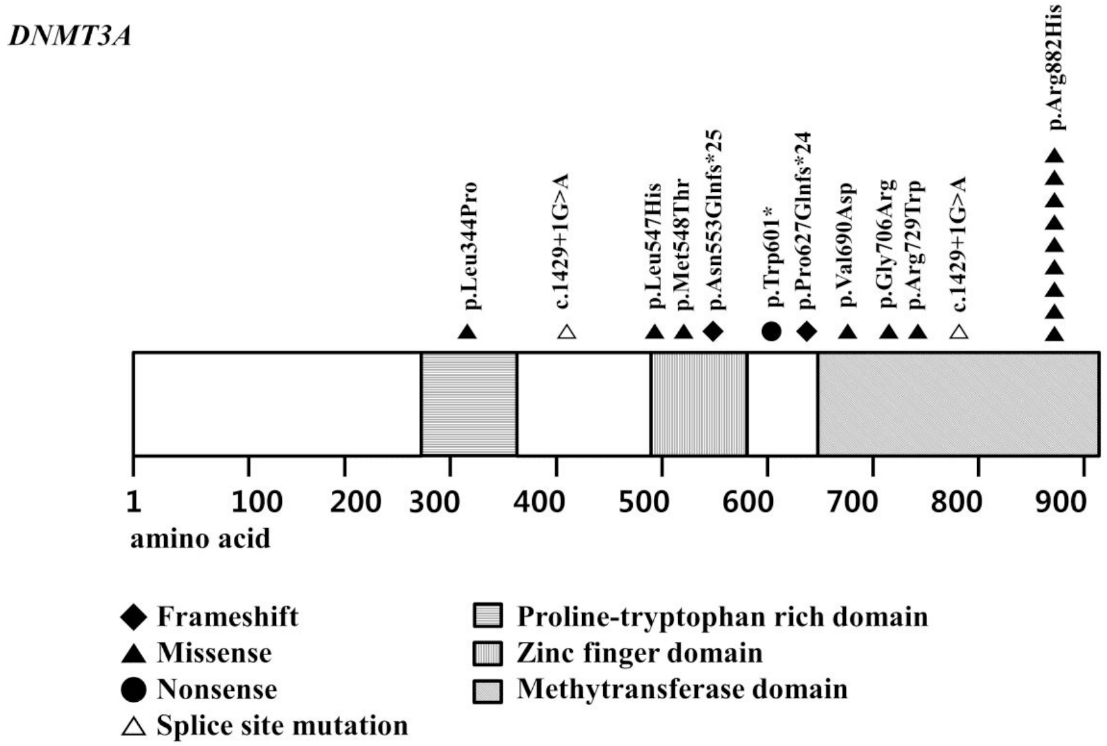

Supplementary Figure S2: Schematic representation of *DNMT3A* gene and location of mutations identified in this study.

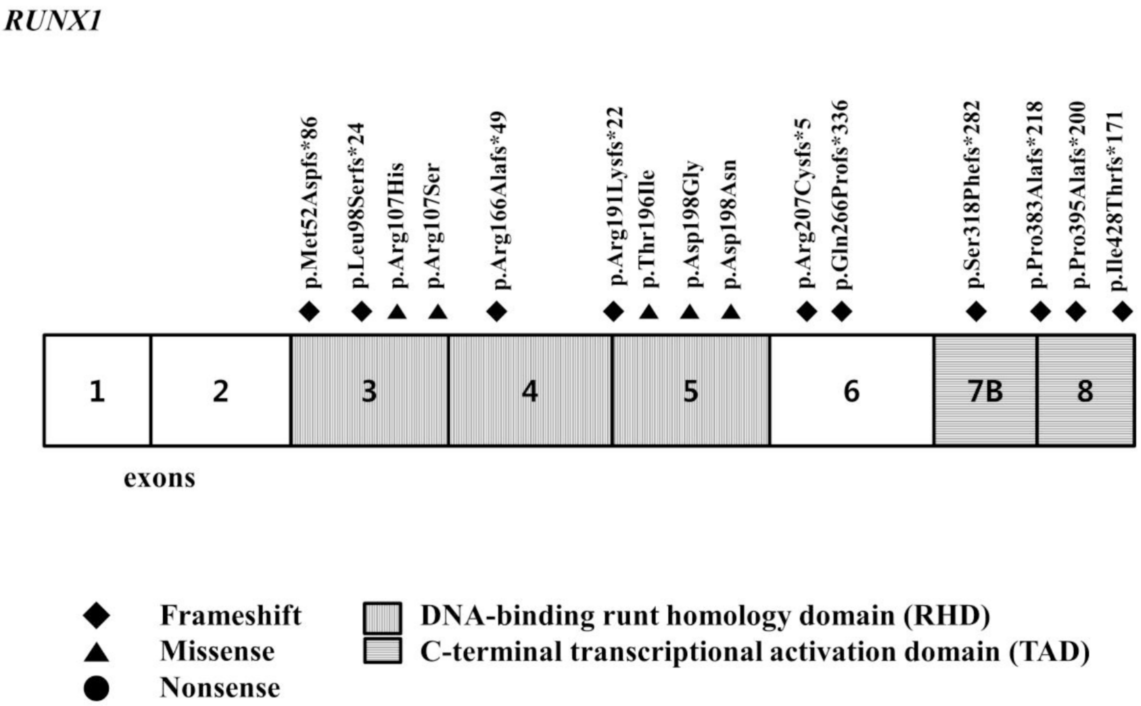

Supplementary Figure S3: Schematic representation of *RUNX1* gene and location of mutations identified in this study.

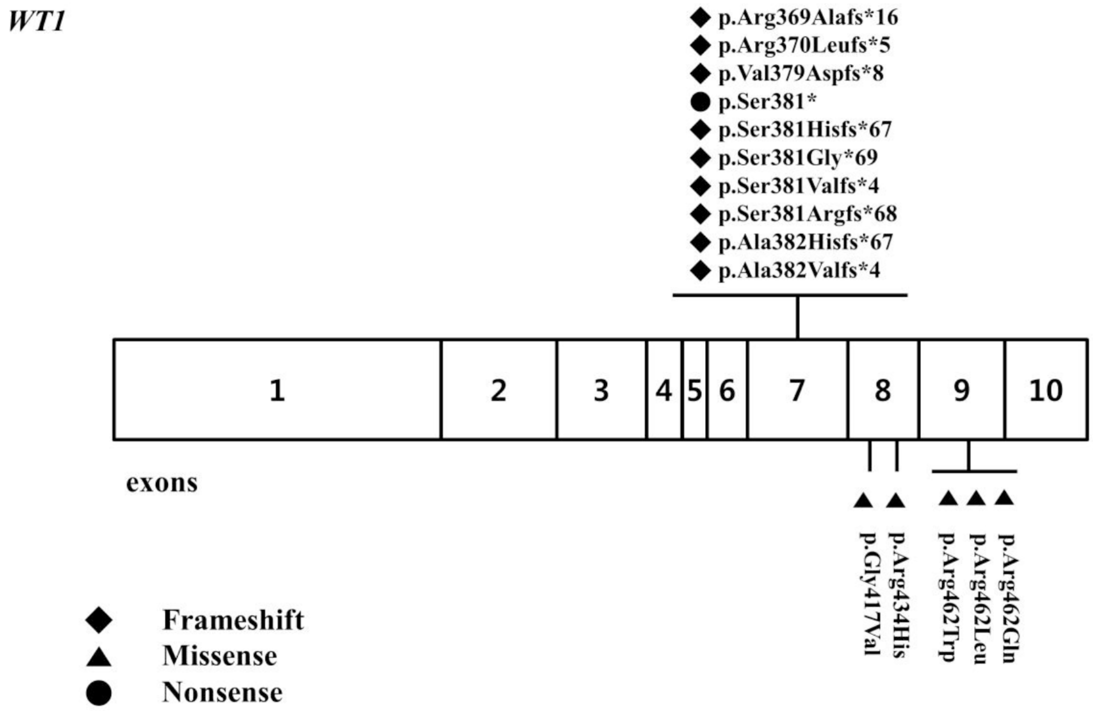

Supplementary Figure S4: Schematic representation of *WT1* gene and location of mutations identified in this study.

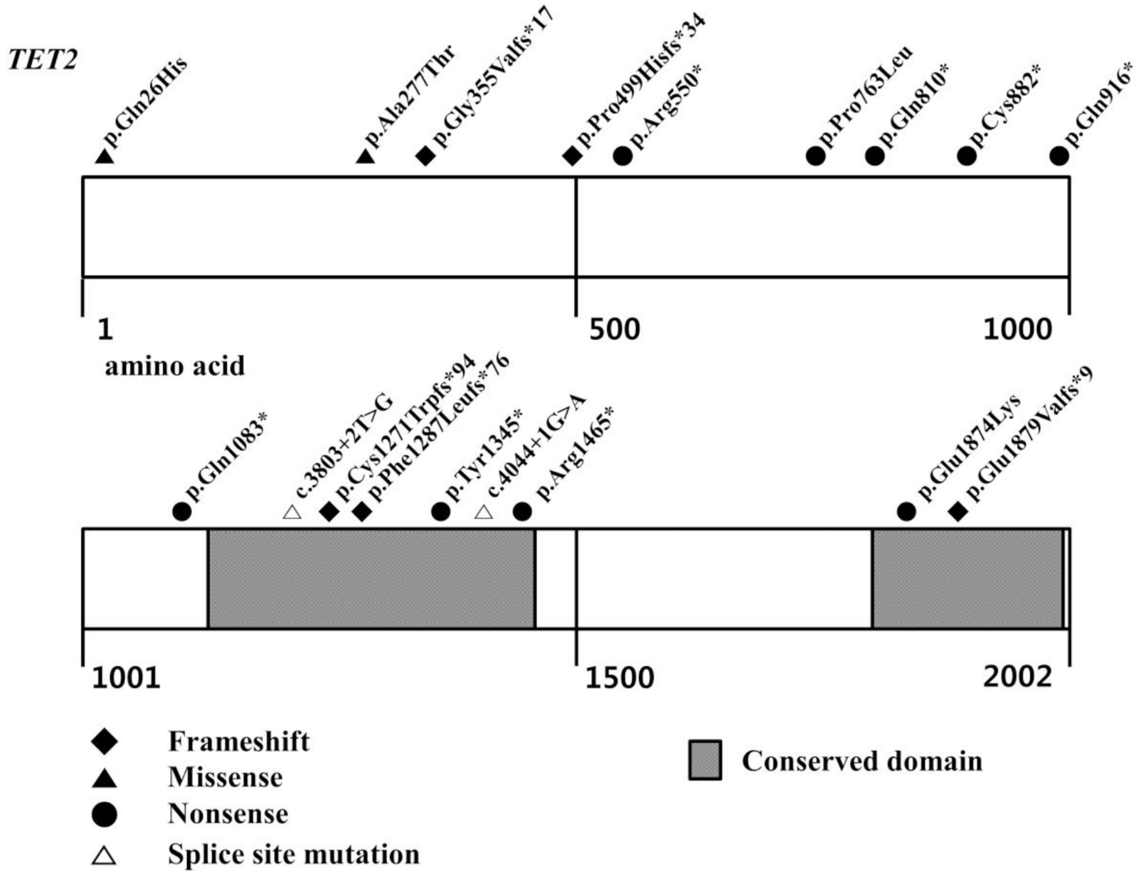

Supplementary Figure S5: Schematic representation of *TET2* gene and location of mutations identified in this study.

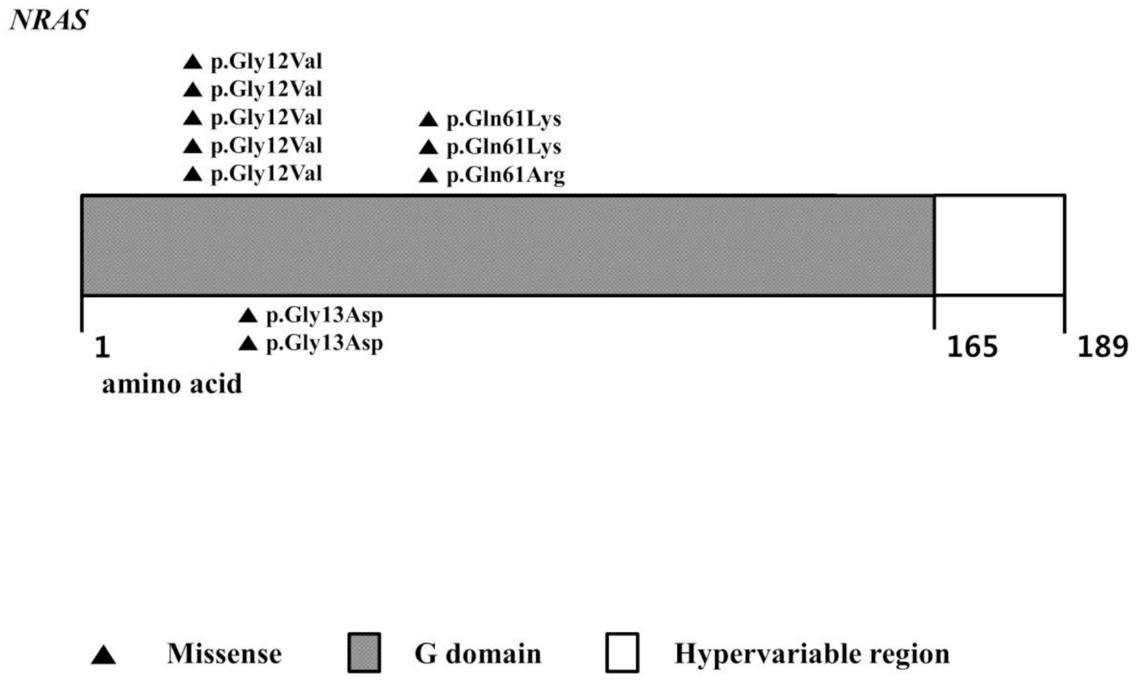

Supplementary Figure S6: Schematic representation of *NRAS* gene and location of mutations identified in this study.

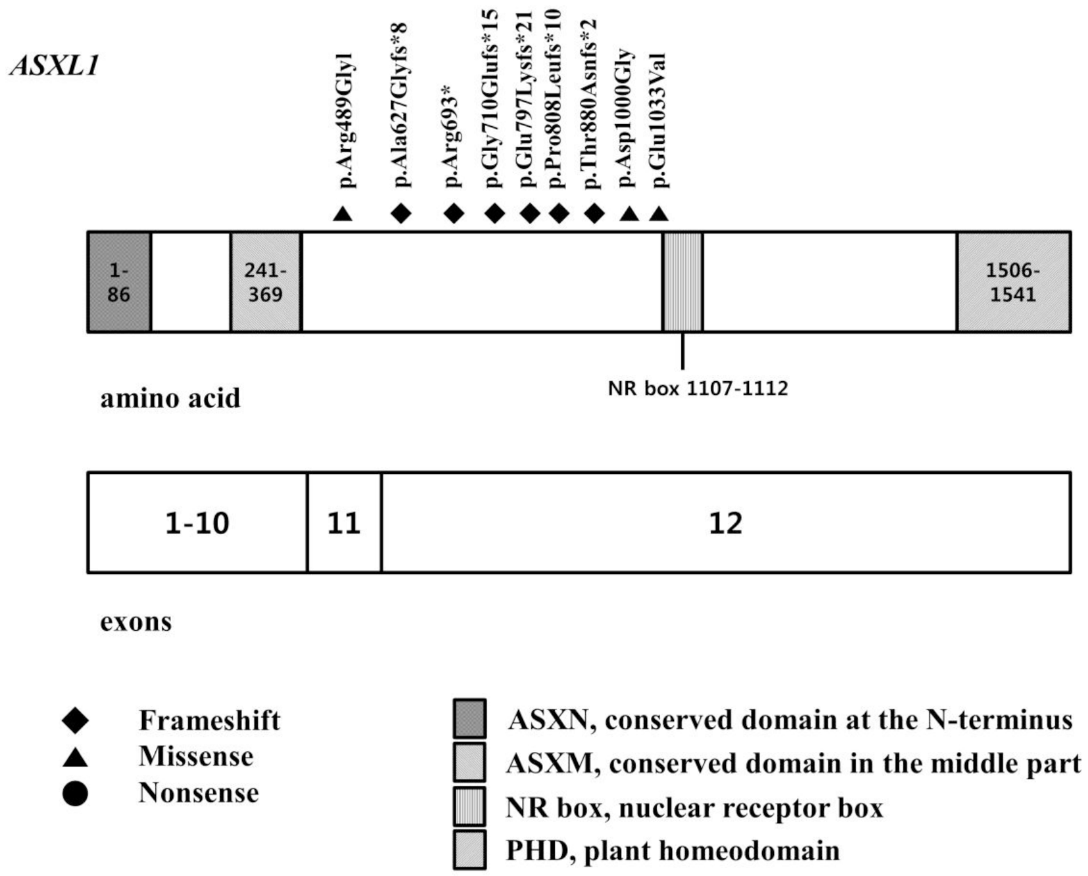

Supplementary Figure S7: Schematic representation of *ASXL1* gene and location of mutations identified in this study.

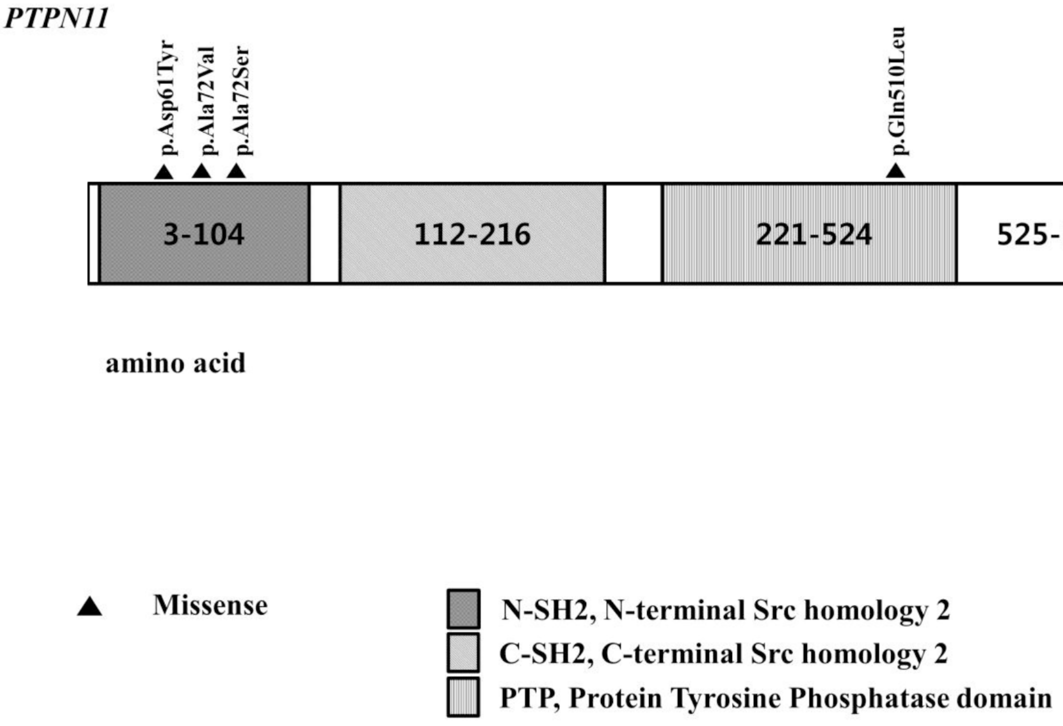

Supplementary Figure S8: Schematic representation of *PTPN11* gene and location of mutations identified in this study.

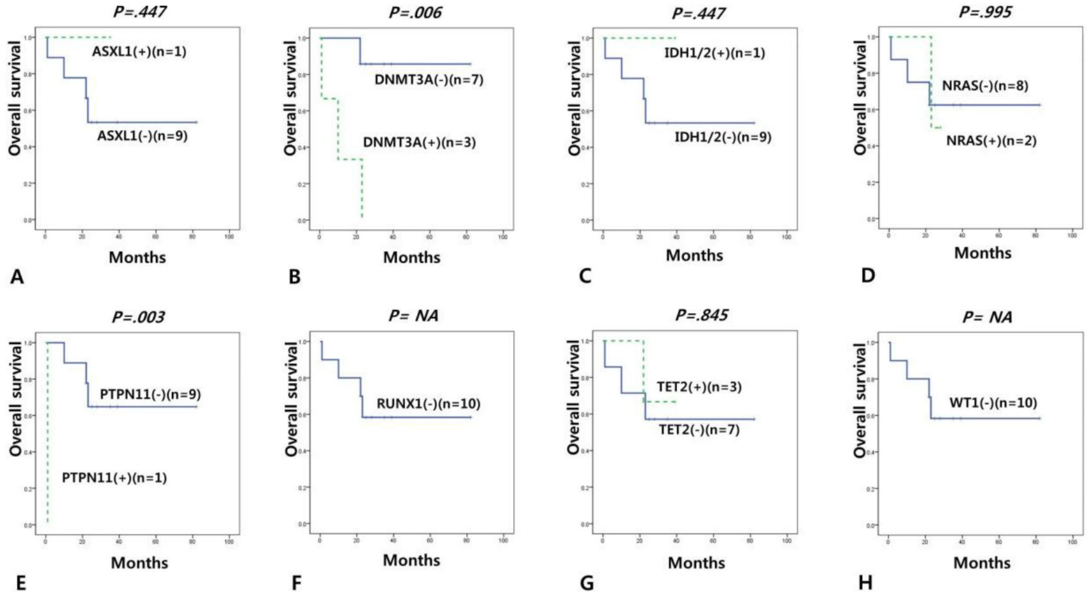

Supplementary Figure S9: Overall survival according to gene mutation in favorable group (adult *de novo* CN-AML with mutated *NPM1* without *FLT3*-ITD or CN-AML with mutated *CEBPA*) of AML defined by ELN.

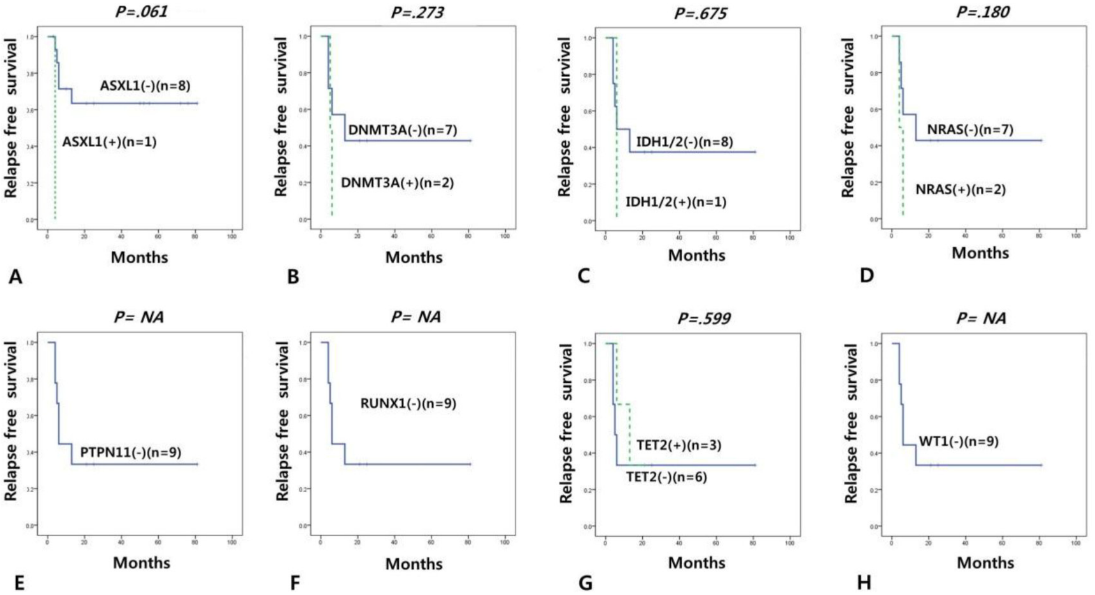

Supplementary Figure S10: Relapse free survival according to gene mutations in favorable group (adult *de novo* CN-AML with mutated *NPM1* without *FLT3*-ITD or CN-AML with mutated *CEBPA*) of AML defined by ELN.

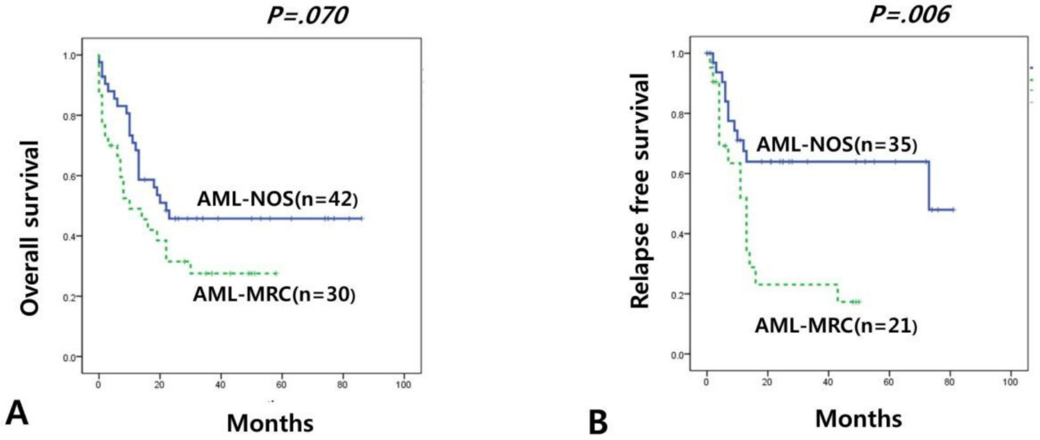

Supplementary Figure S11: Clinical outcomes of *de novo* adult AML-MRC and AML-NOS.

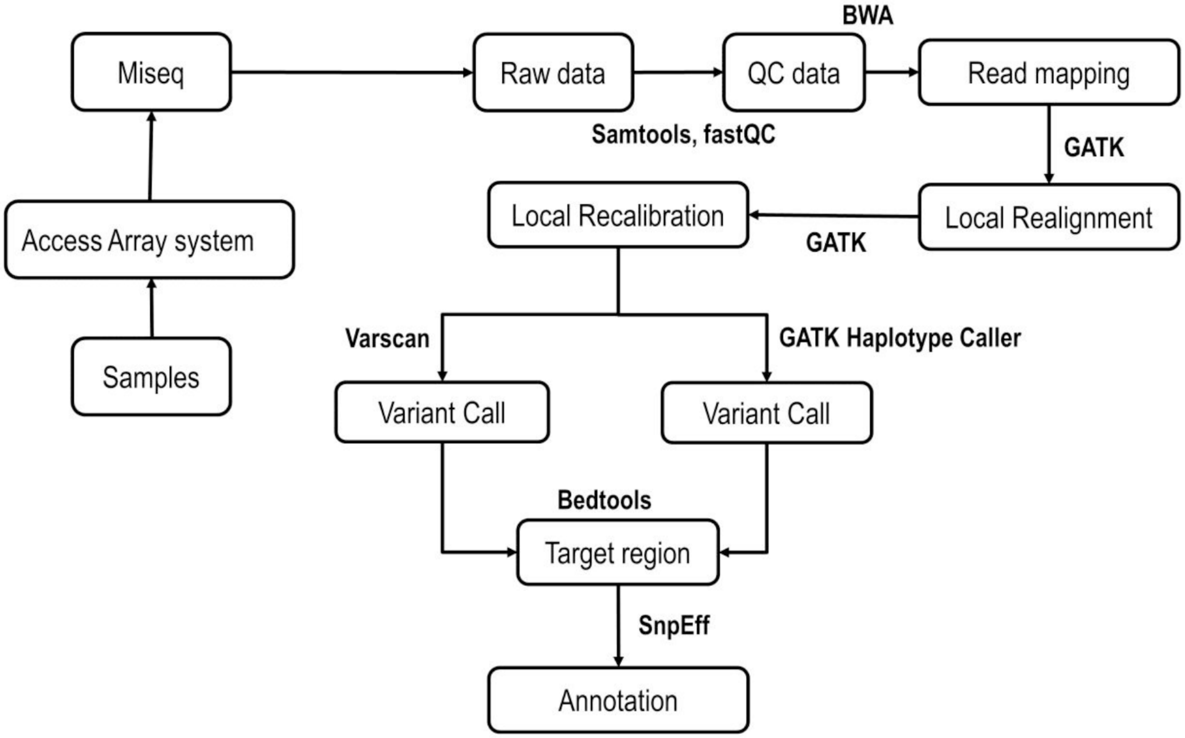

Supplementary Figure S12: Flow chart of data analysis of targeted gene sequencing. SAMtool and fastQC were used for qulity control. The resulting raw files were aligned to the human reference genome (hg19) using the Burrows-Wheeler Aligner (BWA) algorithm with default parameters. Genome Analysis Toolkit (GATK) was used to local alignment. GATK-Haplotype caller and Varscan were used as tools for variant callling. BEDTools was used for estimating read depth and coverage. Variant annoatation was performed using SnpEff.

Supplementary Table S1: Basic characteristics of enrolled subjects.

See Supplementary File 1

Supplementary Table S2: Number of variations filtered from NGS data and confirmed by Sanger sequencing

Gene	Variants identified	Confirmed by Sanger sequencing	Total variations confirmed*
ASXL1	9	9/9(90%)	9
BRAF	0	-	-
CBL	0	-	-
CEBPA	14	10/14(71.4%)	10
DNMT3A	23	20/23(87.0%	20
FLT3-ITD	9	9/9(100%)	34
FLT3-TKD	6	5/6(83.3%)	6
IDH1	9	8/9(88.9%)	8
IDH2	10	10/10(100%)	10
JAK2	2	2/2(100%)	3
KIT	4	2/4(50%)	2
KRAS	1	0/1(0%)	-
NPM1	23	23/23(100%)	24
NRAS	11	10/11(90.9%)	10
PTPN11	6	4/6(66.7%)	4
RUNX1	22	15/22(68.2%)	15
SETD2	8	4/8(50%)	4
TET2	22	17/22(77.3%)	17
TP53	5	3/5(60.0%)	3
WT1	15	15/15(100%)	15

* For *FLT3*-ITD, *FLT3*-TKD, *JAK2* V617F, *KIT, CEBPA* and *NPM1* mutations, we adopted and integrated results of clinical testing wherever available

Supplementary Table S3: Mutations identified and confirmed by Sanger sequencing.

See Supplementary File 2

Supplementary Table S4: Relapse and complete remission rate according to gene mutations (adult *de novo* AML)

		complete remission		***P***	relapse		***P***
		**-**	**+**		**-**	**+**	
FLT3-ITD	-	3	41	.546	20	21	.205
	+	0	23		15	8	
NPM1	-	3	47	.565	26	21	.866
	+	0	17		9	8	
DNMT3A	-	3	54	1.000	33	21	.034
	+	0	10		2	8	
RUNX1	-	0	60	.001	31	29	.120
	+	3	4		4	0	
WT1	-	2	60	.211	31	29	.120
	+	1	4		4	0	
TET2	-	2	55	.389	30	25	1.000
	+	1	9		5	4	
IDH1/2	-	3	54	1.000	30	24	1.000
	+	0	10		5	5	
NRAS	-	3	60	1.000	35	25	.037
	+	0	4		0	4	
ASXL1	-	3	57	1.000	31	26	1.000
	+	0	7		4	3	

Supplementary Table S5: Multivariable cox regression analysis for overall survival (adult AML)

	CN-AML, *de novo* (n=66)				Intermediate cytogenetic risk group, * *de novo* (n=68)			
	P	Hazard ratio	95.0% CI		*P*	Hazard ratio	95.0% CI	
			Lower	Upper			Lower	Upper
Age >60	.428	1.361	.635	2.916	.270	1.538	.716	3.304
Sex	.728	.872	.403	1.887	.943	.973	.461	2.054
FLT3-ITD	.601	.824	.400	1.700	.815	.918	.446	1.887
NPM1	.854	.928	.419	2.054	.875	.938	.424	2.076
DNMT3A	.002	4.042	1.646	9.923	.003	3.762	1.558	9.081
RUNX1	.077	2.804	.894	8.798	.220	2.002	.660	6.074
WT1	.337	1.789	.545	5.866	.189	2.154	.685	6.775
TET2	.913	1.049	.445	2.471	.779	1.130	.480	2.661
IDH1/2	.473	.717	.289	1.779	.562	.766	.312	1.885

* Normal karyotype, t(9;11)(p22;q23) and cytogenetic abnormalities classified neither as favorable nor adverse by the ELN system

Supplementary Table S6: Multivariable cox regression analysis for relapse free survival (adult AML)

	CN-AML, *de novo* (n=53)				Intermediate cytogenetic risk group, * *de novo* (n=55)			
	P	Hazard ratio	95.0% CI		*P*	Hazard ratio	95.0% CI	
			Lower	Upper			Lower	Upper
Age >60	.453	1.393	.586	3.312	.335	1.535	.643	3.664
Sex	.642	.806	.324	2.005	.712	.843	.340	2.089
FLT3-ITD	.650	.817	.341	1.957	.772	.879	.368	2.102
NPM1	.789	1.135	.448	2.877	.943	1.035	.406	2.634
DNMT3A	.011	4.553	1.423	14.574	.012	4.554	1.400	14.810
RUNX1	.989	.000	0.000	-	.989	1922559.183	0.000	-
WT1	.983	.000	0.000	-	.988	.000	0.000	-
TET2	.535	.678	.198	2.318	.592	.714	.209	2.443
IDH1/2	.101	.367	.111	1.215	.112	.376	.112	1.255

* Normal karyotype, t(9;11)(p22;q23) and cytogenetic abnormalities classified neither as favorable nor adverse by the ELN system

Supplementary Table S7: Genes and exons sequenced

Gene	Exon numbers
TET2	All
DNMT3A	All
IDH1	4
IDH2	4
NPM1	10,11
FLT3	15,20
CEBPA	1
ASXL1	11,12
BRAF	11,15
CBL	7,8,9
KIT	8,9,10,11,17
KRAS	1,2,3,4
NRAS	1,2,3,4
PTPN11	3,7,8,12,13
RUNX1	3,4,5,6,7,8
TP53	3,4,5,6,8,9
WT1	4,5,6,7,8,9
SETD2	All
JAK2	12,14

Supplementary Table S8: Primers used for Sanger sequencing.

See Supplementary File 3
