# Supplementary material for: Mutation profiling of 19 candidate genes in acute myeloid leukemia suggests significance of DNMT3A mutations
Source: Oncotarget. 2016 Jun 23;7(34):54825–37. doi: 10.18632/oncotarget.10240 (PMC5342384; doi:10.18632/oncotarget.10240)
Supplement: Supplementary file 2 [file oncotarget-07-54825-s002.docx]

**Supplementary Table S1**. **Basic characteristics of enrolled subjects**

| **No.** | **Age** | **Sex** | **Chromosome** | **Diagnosis** |
| --- | --- | --- | --- | --- |
| hema1-22_S22 | 45 | F | 47,XX,+9[8]/46,XX[12] | M1 |
| hema1-23_S23 | 6 | M | 46,XY,inv(11)(p15q23)[11]/46,XY[9] | M4 |
| hema1-24_S24 | 3 | M | 46,XY[20] | M2 |
| hema1-25_S25 | 66 | M | 46,XY,del(20)(q11.20)[20] | MRC |
| hema1-26_S26 | 67 | M | 46,XX,t(8;21)(q22;q22)[18]/46,XX[2] | AML with t(8;21)(q22;q22) |
| hema1-28_S28 | 71 | M | NA | NA |
| hema1-29_S29 | 59 | M | 42,XY,add(3)(p24),-4,-5,-7,add(11)(p15),add(12)(p12),-13,del(20)(q13.1)[13]/42,sl,del(1)(q32)[5]/46,XY[2] | AML, MRC |
| hema1-30_S30 | 51 | M | No mitotic cells | M2 |
| hema1-31_S31 | 60 | M | NA | NA |
| hema1-32_S32 | 71 | F | 46,XX[20] | AML, MRC |
| hema1-34_S34 | 71 | F | NA | NA |
| hema1-35_S35 | 70 | M | 46,XY[16] | M5 |
| hema1-36_S36 | 72 | F | NA | NA |
| hema1-38_S38 | 87 | F | NA | NA |
| hema1-39_S39 | 72 | F | NA | NA |
| hema1-40_S40 | 8 | F | 46,XX,t(8;21)(q22;q22)[14]/46,XX[6] | AML with t(8;21)(q22;q22) |
| hema2-11_S11 | 55 | M | 46,XY,inv(16)(p13.1q22)[11]/46,XY[2] | AML with inv(16)(p13q22) |
| hema2-12_S12 | 52 | M | 46,XX[11] | MRC |
| hema2-13_S13 | 39 | M | 46,XY[20] | M2 |
| hema2-14_S14 | 30 | F | 45,X,-X,t(8;21)(q22;q22)[18]/46,XX[2] | AML with t(8;21)(q22;q22) |
| hema2-15_S15 | 63 | F | 49,XX,del(13)(q12q14),+del(13),+14,+19[4] | AML, MRC |
| hema2-16_S16 | 45 | M | 46,XY[40] | M2 |
| hema2-17_S17 | 48 | M | 48,XY,+der(1)t(1;19)(p13;p13.1),+9[14]/46,XY[6] | M1 |

**Supplementary Table S1.** ***Continued.***

| **No.** | **Age** | **Sex** | **Chromosome** | **Diagnosis** |
| --- | --- | --- | --- | --- |
| hema2-19_S19 | 55 | M | 46,XY[20] | AML with  t(15;17)(q22;q21) |
| hema2-20_S20 | 53 | M | 46,XY[20] | M2 |
| hema2-21_S21 | 8 | M | 46,XY,+del(1)(q21),der(1;18)(q10;q10),del(10)(p13)[19]//46,XX[1] | AML, MRC |
| hema2-22_S22 | 62 | M | 45,XY,add(3)(p21),-8,add(11)(p15),der(17;18)(q10;q10),+mar[12] /45,sl,del(9)(q13q22)[8] | MRC |
| hema2-23_S23 | 6 | F | 46,XX[20] | M5 |
| hema2-24_S24 | 6 | M | 42,X,-Y,add(5)(q13),-6,add(7)(q22),add(11)(p15)x2, +add(11)(q25),der(13)t(13;15)(p11.2;q15),-15,-17,-17[13]/41, sl,add(3)(q12),del(5)(q32),-add(11)(p15),+mar[7] | MRC |
| hema2-25_S25 | 15 | M | 47,XY,+8[14]/46,XY[3] | M5 |
| hema2-26_S26 | 74 | M | 92,XXYY[4]/89,XXYY,-2,del(7)(q22),-9,-17[2]/46,XY[14] | MRC |
| hema2-27_S27 | 37 | F | 46,XX[20] | M2 |
| hema2-28_S28 | 59 | M | 46,XY[10] | M2 |
| hema2-29_S29 | 100 | F | 48,XX,+19,+19[19]/46,XX[1] | M0 |
| hema2-30_S30 | 50 | F | 46,XX[20] | AML,MRC |
| hema2-31_S31 | 66 | M | 46,XY[12] | AML, MRC |
| hema2-32_S32 | 9 | F | 46,XX,t(8;21)(q22;q22)[3]/45,sl,-X[14] | AML with t(8;21)(q22;q22) |
| hema2-33_S33 | 37 | M | 46,XY[17] | AML,MRC |
| hema2-34_S34 | 56 | M | 46,XX,t(11;19)(q23;p13.1)[12]/51,sl,  +5,+13,+13,+19,+21[8] | AML, MRC |
| hema2-35_S35 | 40 | M | 46,XY[17]//46,XX[3] | M7 |
| hema2-36_S36 | 18 | F | 46,XX,der(16)inv(16)(p13.1q22)t(11;16)(q13;q24)[20] | AML with inv(16)(p13q22) |
| hema2-37_S37 | 41 | M | 46,XY[20] | M2 |
| hema2-38_S38 | 58 | F | 46,XX,t(15;17)(q22;q21)[14]/46,XX[6] | AML with  t(15;17)(q22;q21) |
| hema2-39_S39 | 2 | F | 53,XX,+X,+X,+der(1;2)(q10;q10),+6,add(6)(q23)x2,der(7;12) (q10;q10)del(12)(q24.1),+del(8)(q24.1),  +10,+19,+mar[20] | MRC |

**Supplementary Table S1.** ***Continued.***

| **No.** | **Age** | **Sex** | **Chromosome** | **Diagnosis** |
| --- | --- | --- | --- | --- |
| hema2-40_S40 | 61 | M | 50,XY,+der(1;7)(q10;p10),+8,+13,+13[20] | AML, MRC |
| hema2-41_S41 | 41 | M | 45,XY,der(2)t(2;6)(q34;p12),add(6)(q14),-7,-11,+mar[16]/46,XY[4] | AML, MRC |
| hema2-42_S42 | 64 | M | 46,XY[20] | AML, MRC |
| hema3-1_S1 | 65 | F | NA | NA |
| hema3-2_S2 | 45 | M | NA | NA |
| hema3-3_S3 | 32 | M | NA | NA |
| hema3-4_S4 | 71 | F | 45,XX,-7[18]/46,XX[2] | AML, MRC |
| hema3-5_S5 | 53 | M | NA | NA |
| hema3-6_S6 | 48 | F | 46,XX[20] | AML, MRC |
| hema3-8_S8 | 70 | M | 46,XY,der(4)t(4;10)(q35;q11.2)[20] | AML. MRC |
| hema3-23_S23 | 43 | F | 46,XX[15] | M1 |
| hema3-25_S25 | 58 | F | 46,XX[7] | M2 |
| hema3-26_S26 | 65 | F | 46,XX[20] | M2 |
| hema3-27_S27 | 64 | F | 46,XX[20] | MRC |
| hema3-28_S28 | 55 | M | 46,XY[20] | M2 |
| hema3-29_S29 | 45 | M | 46,XY[15] | M0 |
| hema3-30_S30 | 60 | M | 46,XY[20] | AML, MRC |
| hema3-31_S31 | 11 | M | 46,XY[16] | MRC |
| hema3-32_S32 | 3 | F | 46,XX[20] | M6 |
| hema3-33_S33 | 1 | F | 46,XX[20] | M7 |
| hema3-34_S34 | 68 | M | 46,XY[20] | M1 |
| hema3-35_S35 | 67 | F | 46,XX[20] | M4 |
| hema3-36_S36 | 52 | F | 46,XX[9] | AML, MRC |
| hema3-37_S37 | 34 | F | 46,XX[20] | M2 |
| hema3-38_S38 | 71 | F | 46,XX[8] | AML, MRC |

**Supplementary Table S1.** ***Continued.***

| **No.** | **Age** | **Sex** | **Chromosome** | **Diagnosis** |
| --- | --- | --- | --- | --- |
| hema3-39_S39 | 31 | M | 46,XY[20] | M2 |
| hema3-40_S40 | 66 | M | 46,XY[20] | AML, MRC |
| hema3-41_S41 | 65 | M | 46,XY[17] | AML, MRC |
| hema3-42_S42 | 62 | M | 46,XY[20] | M2 |
| hema3-43_S43 | 55 | F | 46,XX[20] | AML, MRC |
| hema3-44_S44 | 63 | M | 46,XY[20] | AML, MRC |
| hema3-45_S45 | 3 | M | 46,XY[20] | AML, MRC |
| hema3-46_S46 | 34 | M | 46,XY[20] | AML, MRC |
| hema4-1_S1 | 73 | F | 46,XX[2] | AML, MRC |
| hema4-2_S2 | 58 | M | 46,XY[20] | AML, MRC |
| hema4-3_S3 | 57 | M | 46,XY[20] | AML, MRC |
| hema4-4_S4 | 78 | M | 46,XY[20] | M4 |
| hema4-5_S5 | 84 | M | 46,XY[20] | AML, MRC |
| hema4-6_S6 | 72 | M | 46,XY[20] | M4 |
| hema4-7_S7 | 60 | F | 46,XX[20] | AML, MRC |
| hema4-8_S8 | 43 | F | 46,XX[20] | AML, MRC |
| hema4-9_S9 | 58 | M | 46,XY[20] | M2 |
| hema4-10_S10 | 44 | F | 46,XX[20] | M4 |
| hema4-11_S11 | 20 | M | 46,XY[20] | M4 |
| hema4-12_S12 | 50 | M | 46,XY[20] | AML, MRC |
| hema4-13_S13 | 38 | F | 46,XX[20] | M4 |
| hema4-14_S14 | 69 | M | 46,XY[20] | M2 |
| hema4-15_S15 | 74 | F | 46,XX[20] | M0 |
| hema4-16_S16 | 64 | M | 46,XY[20] | M1 |
| hema4-17_S17 | 59 | M | 46,XY[20] | AML, MRC |

**Supplementary Table S1.**. ***Continued.***

| **No.** | **Age,** | **Sex** | **Chromosome** | **Diagnosis** |
| --- | --- | --- | --- | --- |
| hema4-18_S18 | 61 | F | 46,XX[20] | M1 |
| hema4-19_S19 | 67 | M | 46,XY[20] | M0 |
| hema4-20_S20 | 56 | F | 46,XX[20] | M1 |
| hema4-21_S21 | 55 | F | 46,XX[20] | M1 |
| hema4-22_S22 | 19 | M | 46,XY[20] | M6 |
| hema4-23_S23 | 54 | M | 46,XY[20] | M4 |
| hema4-24_S24 | 68 | M | 46,XY[20] | M2 |
| hema4-25_S25 | 73 | M | 46,XY[20] | M4 |
| hema4-26_S26 | 45 | M | 46,XY[20] | M2 |
| hema4-27_S27 | 70 | F | 46,XX[20] | AML, MRC |
| hema4-28_S28 | 67 | M | 45,X,-Y[12]/46,XY[8] | M0 |
| hema4-29_S29 | 52 | F | 46,XX[20] | M1 |
| hema4-30_S30 | 54 | M | 46,XY[5] | AML, MRC |
| hema4-31_S31 | 71 | F | 46,XX[20] | AML, MRC |
| hema4-32_S32 | 34 | M | 46,XY[15] | M4 |
| hema4-33_S33 | 26 | M | 46,XY[20] | AML, MRC |
| hema4-34_S34 | 61 | M | 46,XY[20] | M2 |
| hema4-35_S35 | 65 | M | 46,XY[20] | M2 |
| hema4-36_S36 | 41 | M | 46,XY[20] | M2 |
| hema4-37_S37 | 58 | F | 46,XX[20] | M1 |
