# Supplementary material for: Mutation profiling of 19 candidate genes in acute myeloid leukemia suggests significance of DNMT3A mutations
Source: Oncotarget. 2016 Jun 23;7(34):54825–37. doi: 10.18632/oncotarget.10240 (PMC5342384; doi:10.18632/oncotarget.10240)
Supplement: Supplementary file 3 [file oncotarget-07-54825-s003.docx]

**Supplementary Table S3.** **Mutations identified and confirmed by Sanger sequencing**

| **Patients** | **Gene** | **Exon number** | **cDNA** | **Protein** |
| --- | --- | --- | --- | --- |
| hema1-24_S24 | *NPM1* | 11 | NM_002520.6:c.863_864insCATG | p.Trp288Cysfs*12 |
| hema1-34_S34 | *NPM1* | 11 | NM_002520.6:c.860_863dupTCTG | p.Trp288Cysfs*12 |
| hema3-6_S6 | *NPM1* | 11 | NM_002520.6:c.860_863dupTCTG | p.Trp288Cysfs*12 |
| hema3-25_S25 | *NPM1* | 11 | NM_002520.6:c.860_863dupTCTG | p.Trp288Cysfs*12 |
| hema3-27_S27 | *NPM1* | 11 | NM_002520.6:c.860_863dupTCTG | p.Trp288Cysfs*12 |
| hema3-35_S35 | *NPM1* | 11 | NM_002520.6:c.860_863dupTCTG | p.Trp288Cysfs*12 |
| hema3-40_S40 | *NPM1* | 11 | NM_002520.6:c.860_863dupTCTG | p.Trp288Cysfs*12 |
| hema3-41_S41 | *NPM1* | 11 | NM_002520.6:c.863_864insCCTG | p.Trp288Cysfs*12 |
| hema3-45_S45 | *NPM1* | 11 | NM_002520.6:c.860_863dupTCTG | p.Trp288Cysfs*12 |
| hema4-2_S2 | *NPM1* | 11 | NM_002520.6:c.863_864insCATG | p.Trp288Cysfs*12 |
| hema4-4_S4 | *NPM1* | 11 | NM_002520.6:c.860_863dupTCTG | p.Trp288Cysfs*12 |
| hema4-10_S10 | *NPM1* | 11 | NM_002520.6:c.860_863dupTCTG | p.Trp288Cysfs*12 |
| hema4-16_S16 | *NPM1* | 11 | NM_002520.6:c.860_863dupTCTG | p.Trp288Cysfs*12 |
| hema4-18_S18 | *NPM1* | 11 | NM_002520.6:c.860_863dupTCTG | p.Trp288Cysfs*12 |
| hema4-21_S21 | *NPM1* | 11 | NM_002520.6:c.860_863dupTCTG | p.Trp288Cysfs*12 |
| hema4-24_S24 | *NPM1* | 11 | NM_002520.6:c.863_864insCATG | p.Trp288Cysfs*12 |
| hema4-25_S25 | *NPM1* | 11 | NM_002520.6:c.860_863dupTCTG | p.Trp288Cysfs*12 |
| hema4-27_S27 | *NPM1* | 11 | NM_002520.6:c.860_863dupTCTG | p.Trp288Cysfs*12 |
| hema4-28_S28 | *NPM1* | 11 | NM_002520.6:c.860_863dupTCTG | p.Trp288Cysfs*12 |
| hema4-29_S29 | *NPM1* | 11 | NM_002520.6:c.860_863dupTCTG | p.Trp288Cysfs*12 |
| hema4-31_S31 | *NPM1* | 11 | NM_002520.6:c.860_863dupTCTG | p.Trp288Cysfs*12 |
| hema4-33_S33 | *NPM1* | 11 | NM_002520.6:c.863_864insCCTG | p.Trp288Cysfs*12 |
| hema4-35_S35 | *NPM1* | 11 | NM_002520.6:c.860_863dupTCTG | p.Trp288Cysfs*12 |
| hema4-37_S37 | *NPM1* | 11 | NM_002520.6:c.860_863dupTCTG | p.Trp288Cysfs*12 |
| hema1-34_S34 | *DNMT3A* | 17 | NM_175629.2:c.2069T>A | p.Val690Asp |
| hema1-36_S36 | *DNMT3A* | 23 | NM_175629.2:c.2645G>A | p.Arg882His |

**Supplementary Table S3.** ***Continued*.**

| **Patients** | **Gene** | **Exon number** | **cDNA** | **Protein** |
| --- | --- | --- | --- | --- |
| hema1-38_S38 | *DNMT3A* | 11 | NM_175629.2:c.1429+1G>A | - |
| hema2-12_S12 | *DNMT3A* | 23 | NM_175629.2:c.2645G>A | p.Arg882His |
| hema2-20_S20 | *DNMT3A* | 15 | NM_175629.2:c.1803G>A | p.Trp601* |
| hema3-6_S6 | *DNMT3A* | 16 | NM_175629.2:c.2645G>A | p.Arg882His |
| hema3-27_S27 | *DNMT3A* | 16 | NM_175629.2:c.1880delC | p.Pro627Glnfs*24 |
| hema3-35_S35 | *DNMT3A* | 23 | NM_175629.2:c.2645G>A | p.Arg882His |
| hema3-41_S41 | *DNMT3A* | 14 | NM_175629.2:c.1643T>C | p.Met548Thr |
| hema3-42_S42 | *DNMT3A* | 23 | NM_175629.2:c.2645G>A | p.Arg882His |
| hema3-45_S45 | *DNMT3A* | 14 | NM_175629.2:c.1655_1656insT | p.Asn553Glnfs*25 |
| hema4-4_S4 | *DNMT3A* | 23 | NM_175629.2:c.2645G>A | p.Arg882His |
| hema4-6_S6 | *DNMT3A* | 9 | NM_175629.2:c.2645G>A | p.Arg882His |
| hema4-9_S9 | *DNMT3A* | 19 | NM_175629.2:c.2116G>A | p.Gly706Arg |
| hema4-10_S10 | *DNMT3A* | 23 | NM_175629.2:c.2185C>T | p.Arg729Trp |
| hema4-12_S12 | *DNMT3A* | 23 | NM_175629.2:c.1640T>A | p.Leu547His |
| hema4-24_S24 | *DNMT3A* | 18 | NM_175629.2:c.2645G>A | p.Arg882His |
| hema4-25_S25 | *DNMT3A* | 19 | NM_175629.2:c.2185C>T | p.Arg729Trp |
| hema4-31_S31 | *DNMT3A* | 14 | NM_175629.2:c.1640T>A | p.Leu547His |
| hema4-34_S34 | *DNMT3A* | 23 | NM_175629.2:c.2645G>A | p.Arg882His |
| hema1-22_S22 | *RUNX1* | 6 | NM_001754.4:c.794_795insCCCCGAG | p.Gln266Profs*336 |
| hema1-36_S36 | *RUNX1* | 8 | NM_001754.4:c.1180_1181dup | p.Pro395Alafs*200 |
| hema2-22_S22 | *RUNX1* | 6 | NM_001754.4:c.618_619insTG | p.Arg207Cysfs*5 |
| hema2-26_S26 | *RUNX1* | 3 | NM_001754.4:c.319C>A | p.Arg107Ser |
| hema2-31_S31 | *RUNX1* | 5 | NM_001754.4:c.592G>A | p.Asp198Asn |
| hema2-40_S40 | *RUNX1* | 3 | NM_001754.4:c.320G>A | p.Arg107His |
| hema3-4_S4 | *RUNX1* | 5 | NM_001754.4:c.593A>G | p.Asp198Gly |
| hema3-4_S4 | *RUNX1* | 5 | NM_001754.4:c.587C>T | p.Thr196Ile |
| hema3-8_S8 | *RUNX1* | 3 | NM_001754.4:c.292delC | p.Leu98Serfs*24 |

**Supplementary Table S3.** ***Continued.***

| **Patients** | **Gene** | **Exon number** | **cDNA** | **Protein** |
| --- | --- | --- | --- | --- |
| hema4-11_S11 | *RUNX1* | 8 | NM_001754.4:c.1283_1284delTC | p.Ile428Thrfs*171 |
| hema4-13_S13 | *RUNX1* | 7 | NM_001754.4:c.952dupT | p.Ser318Phefs*282 |
| hema4-14_S14 | *RUNX1* | 8 | NM_001754.4:c.1139_1142dupACCT | p.Pro383Alafs*218 |
| hema4-20_S20 | *RUNX1* | 5 | NM_001754.4:c.571dupA | p.Arg191Lysfs*22 |
| hema4-23_S23 | *RUNX1* | 4 | NM_001754.4:c.495_496insGCCGGGT | p.Arg166Alafs*49 |
| hema4-23_S23 | *RUNX1* | 3 | NM_001754.4:c.153dupG | p.Met52Aspfs*86 |
| hema1-23_S23 | *WT1* | 7 | NM_024426.4:c.1142C>A | p.Ser381* |
| hema2-11_S11 | *WT1* | 8 | NM_024426.4:c.1250G>T | p.Gly417Val |
| hema2-21_S21 | *WT1* | 8 | NM_024426.4:c.1301G>A | p.Arg434His |
| hema2-23_S23 | *WT1* | 9 | NM_024426.4:c.1385G>A | p.Arg462Gln |
| hema2-35_S35 | *WT1* | 9 | NM_024426.4:c.1385G>T | p.Arg462Leu |
| hema2-36_S36 | *WT1* | 7 | NM_024426.4:c.1141delT | p.Ser381Argfs*68 |
| hema3-23_S23 | *WT1* | 9 | NM_024426.4:c.1384C>T | p.Arg462Trp |
| hema3-31_S31 | *WT1* | 7 | NM_024426.4:c.1144delG | p.Ala382HisfsTer67 |
| hema3-31_S31 | *WT1* | 7 | NM_024426.4:c.1129_1135dupACTCTTG | p.Val379Aspfs*8 |
| hema3-37_S37 | *WT1* | 7 | NM_024426.4:c.1141_1144delTCGG | p.Ser381HisfsTer67 |
| hema4-13_S13 | *WT1* | 7 | NM_024426.4:c.1138delinsAG | p.Ser381Valfs*4 |
| hema4-20_S20 | *WT1* | 7 | NM_024426.4:c.1139_1140dupGG | p.Ser381Glyfs*69 |
| hema4-20_S20 | *WT1* | 7 | NM_024426.4:c.1104dupG | p.Arg369Alafs16 |
| hema4-25_S25 | *WT1* | 7 | NM_024426.4:c.1141_1144dupTCGG | p.Ala382Valfs4 |
| hema4-33_S33 | *WT1* | 7 | NM_024426.4:c.1109delG | p.Arg370Leufs*5 |
| hema1-30_S30 | *TET2* | 7 | NM_001127208.2:c.3861delT | p.Phe1287Leufs*76 |
| hema2-20_S20 | *TET2* | 8 | NM_001127208.2:c.4044+1G>A | - |
| hema2-28_S28 | *TET2* | 3 | NM_001127208.2:c.829G>A | p.Ala277Thr |
| hema2-28_S28 | *TET2* | 3 | NM_001127208.2:c.2288C>T | p.Pro763Leu |
| hema2-28_S28 | *TET2* | 8 | NM_001127208.2:c.4035T>G | p.Tyr1345* |
| hema2-28_S28 | *TET2* | 11 | NM_001127208.2:c.5632_5633dupCG | p.Glu1879Valfs*9 |

**Supplementary Table S3. *Continued.***

| **Patients** | **Gene** | **Exon number** | **cDNA** | **Protein** |
| --- | --- | --- | --- | --- |
| hema3-26_S26 | *TET2* | 11 | NM_001127208.2:c.5620G>A | p.Glu1874Lys |
| hema3-34_S34 | *TET2* | 3 | NM_001127208.2:c.1062delA | p.Gly355Valfs*17 |
| hema3-37_S37 | *TET2* | 10 | NM_001127208.2:c.4393C>T | p.Arg1465* |
| hema3-40_S40 | *TET2* | 3 | NM_001127208.2:c.2428C>T | p.Gln810* |
| hema3-40_S40 | *TET2* | 7 | NM_001127208.2:c.3812_3813insGTCCC | p.Cys1271Trpfs*94 |
| hema4-14_S14 | *TET2* | 3 | NM_001127208.2:c.1496delC | p.Pro499Hisfs*34 |
| hema4-16_S16 | *TET2* | 3 | NM_001127208.2:c.78G>T | p.Gln26His |
| hema4-21_S21 | *TET2* | 6 | NM_001127208.2:c.3803+2T>G | - |
| hema4-29_S29 | *TET2* | 3 | NM_001127208.2:c.2746C>T | p.Gln916* |
| hema4-29_S29 | *TET2* | 3 | NM_001127208.2:c.3247C>T | p.Gln1083* |
| hema4-36_S36 | *TET2* | 3 | NM_001127208.2:c.2646C>A | p.Cys882* |
| hema2-40_S40 | *IDH1* | 4 | NM_005896.2:c.394C>T | p.Arg132Cys |
| hema3-43_S43 | *IDH1* | 4 | NM_005896.2:c.395G>A | p.Arg132His |
| hema3-45_S45 | *IDH1* | 4 | NM_005896.2:c.395G>A | p.Arg132His |
| hema4-16_S16 | *IDH1* | 4 | NM_005896.2:c.394C>T | p.Arg132Cys |
| hema4-34_S34 | *IDH1* | 4 | NM_005896.2:c.394C>T | p.Arg132Cys |
| hema4-37_S37 | *IDH1* | 4 | NM_005896.2:c.394C>A | p.Arg132Ser |
| hema4-6_S6 | *IDH1* | 4 | NM_005896.2:c.395G>A | p.Arg132His |
| hema4-9_S9 | *IDH1* | 4 | NM_005896.2:c.394C>T | p.Arg132Cys |
| hema2-12_S12 | *IDH2* | 4 | NM_002168.2:c.515G>A | p.Arg172Lys |
| hema2-26_S26 | *IDH2* | 4 | NM_002168.2:c.419G>A | p.Arg140Gln |
| hema2-40_S40 | *IDH2* | 4 | NM_002168.2:c.424A>C | p.Ile142Leu |
| hema2-42_S42 | *IDH2* | 4 | NM_002168.2:c.419G>A | p.Arg140Gln |
| hema3-42_S42 | *IDH2* | 4 | NM_002168.2:c.515G>A | p.Arg172Lys |
| hema4-10_S10 | *IDH2* | 4 | NM_002168.2:c.419G>A | p.Arg140Gln |
| hema4-12_S12 | *IDH2* | 4 | NM_002168.2:c.419G>A | p.Arg140Gln |
| hema4-18_S18 | *IDH2* | 4 | NM_002168.2:c.419G>A | p.Arg140Gln |

**Supplementary Table S3.** ***Continued.***

| **Patients** | **Gene** | **Exon number** | **cDNA** | **Protein** |
| --- | --- | --- | --- | --- |
| hema4-5_S5 | *IDH2* | 4 | NM_001127208.2:c.5620G>A | p.Glu1874Lys |
| hema4-8_S8 | *IDH2* | 4 | NM_001127208.2:c.1062delA | p.Gly355Valfs*17 |
| hema2-16_S16 | *CEBPA* | 1 | NM_004364.3:c.225delC | p.Asp75GlufsTer85 |
| hema2-17_S17 | *CEBPA* | 1 | NM_004364.3:c.937_939dupAAG | p.Lys313dup |
| hema2-27_S27 | *CEBPA* | 1 | NM_004364.3:c.933_940delinsGAAAACCC | p. Gln312_Val314delinsLysThrLeu |
| hema2-30_S30 | *CEBPA* | 1 | NM_004364.3:c.929_930insTCT | p.Thr310_Gln311insLeu |
| hema3-23_S23 | *CEBPA* | 1 | NM_004364.3:c.912_913insTTG | p.Lys304_Gln305insLeu |
| hema3-34_S34 | *CEBPA* | 1 | NM_004364.3:c.933delinsCTGC | p.Gln311delinsHisCys |
| hema3-37_S37 | *CEBPA* | 1 | NM_004364.3:c.928A>C | p.Thr310Pro |
| hema3-39_S39 | *CEBPA* | 1 | NM_004364.3:c.929_934delCGCAGC | p.Thr310_Gln312delinsLys |
| hema3-44_S44 | *CEBPA* | 1 | NM_004364.3:c.937_939dupAAG | p.Lys313dup |
| hema4-36_S36 | *CEBPA* | 1 | NM_004364.3:c.898C>G | p.Arg300Gly |
| hema4-36_S36 | *CEBPA* | 1 | NM_004364.3:c.335_343delinsG | p.Pro112Argfs*55 |
| hema1-29_S29 | *NRAS* | 3 | NM_002524.4:c.181C>A | p.Gln61Lys |
| hema1-35_S35 | *NRAS* | 3 | NM_002524.4:c.181C>A | p.Gln61Lys |
| hema2-21_S21 | *NRAS* | 2 | NM_002524.4:c.35G>A | p.Gly12Asp |
| hema2-34_S34 | *NRAS* | 2 | NM_002524.4:c.35G>A | p.Gly12Asp |
| hema3-31_S31 | *NRAS* | 3 | NM_002524.4:c.182A>G | p.Gln61Arg |
| hema3-31_S31 | *NRAS* | 2 | NM_002524.4:c.38G>A | p.Gly13Asp |
| hema4-24_S24 | *NRAS* | 2 | NM_002524.4:c.35G>C | p.Gly12Ala |
| hema4-27_S27 | *NRAS* | 2 | NM_002524.4:c.35G>A | p.Gly12Asp |
| hema4-31_S31 | *NRAS* | 2 | NM_002524.4:c.38G>A | p.Gly13Asp |
| hema4-7_S7 | *NRAS* | 2 | NM_002524.4:c.35G>T | p.Gly12Val |
| hema2-26_S26 | *ASXL1* | 12 | NM_015338.5:c.1879dupG | p.Ala627Glyfs*8 |
| hema2-38_S38 | *ASXL1* | 11 | NM_015338.5:c.1465C>G | p.Arg489Gly |
| hema3-34_S34 | *ASXL1* | 12 | NM_015338.5:c.2999A>G | p.Asp1000Gly |
| hema3-42_S42 | *ASXL1* | 12 | NM_015338.5:c.2077C>T | p.Arg693* |

**Supplementary Table S3.** ***Continued.***

| **Patients** | **Gene** | **Exon number** | **cDNA** | **Protein** |
| --- | --- | --- | --- | --- |
| hema3-6_S6 | *ASXL1* | 12 | NM_015338.5:c.3098A>T | p.Glu1033Val |
| hema3-8_S8 | *ASXL1* | 12 | NM_015338.5:c.2423delC | p.Pro808Leufs*10 |
| hema4-23_S23 | *ASXL1* | 12 | NM_015338.5:c.2129delG | p.Gly710Glufs*15 |
| hema4-2_S2 | *ASXL1* | 12 | NM_015338.5:c.2638dupA | p.Glu797Lysfs*21 |
| hema4-9_S9 | *ASXL1* | 12 | NM_015338.5:c.2638*>+A | p.Thr880Asnfs*2 |
| hema2-38_S38 | *FLT3* | 20 | NM_004119.2:c.2503G>T | p.Asp835Tyr |
| hema3-3_S3 | *FLT3* | 20 | NM_004119.2:c.2503G>T | p.Asp835Tyr |
| hema4-4_S4 | *FLT3* | 20 | NM_004119.2:c.2503G>T | p.Asp835Tyr |
| hema4-13_S13 | *FLT3* | 20 | NM_004119.2:c.2503G>T | p.Asp835Tyr |
| hema4-16_S16 | *FLT3* | 20 | NM_004119.2:c.2505T>A | p.Asp835Tyr |
| hema4-20_S20 | *FLT3* | 20 | NM_004119.2:c.2503G>T | p.Asp835Tyr |
| hema1-24_S24 | *SETD2* | 1 | NM_014159.6:c.44delT | p.Phe15Serfs*96 |
| hema2-29_S29 | *SETD2* | 3 | NM_014159.6:c.3127G>A | p.Glu1043Lys |
| hema3-38_S38 | *SETD2* | 10 | NM_014159.6:c.5155delC | p.Leu1719* |
| hema3-38_S38 | *SETD2* | 3 | NM_014159.6:c.2851A>G | p.Asn951Asp |
| hema2-31_S31 | *PTPN11* | 13 | NM_002834.3:c.1529A>T | p.Gln510Leu |
| hema2-40_S40 | *PTPN11* | 3 | NM_002834.3:c.181G>T | p.Asp61Tyr |
| hema3-27_S27 | *PTPN11* | 3 | NM_002834.3:c.214G>T | p.Ala72Ser |
| hema3-32_S32 | *PTPN11* | 3 | NM_002834.3:c.215C>T | p.Ala72Val |
| hema1-29_S29 | *TP53* | 4 | NM_000546.5:c.334_346delGGCTTCTTGCATT | p.Gly112Leufs*7 |
| hema2-22_S22 | *TP53* | 8 | NM_000546.5:c.818G>A | p.Arg273His |
| hema2-41_S41 | *TP53* | 7 | NM_000546.5:c.716A>G | p.Asn239Ser |
| hema2-28_S28 | *JAK2* | 14 | NM_004972.3:c.1849G>T | p.Val617Phe |
| hema3-30_S30 | *JAK2* | 14 | NM_004972.3:c.1849G>T | p.Val617Phe |
| hema4-29_S29 | *JAK2* | 14 | NM_004972.3:c.1849G>T | p.Val617Phe |

**Supplementary Table S3**. ***Continued.***

| **Patients** | **Gene** | **Exon number** | **cDNA** | **Protein** |
| --- | --- | --- | --- | --- |
| hema1-40_S40 | *KIT* | 17 | NM_001093772.1:c.2434G>C | p.Asp812His |
| hema3-8_S8 | *KIT* | 17 | NM_001093772.1:c.2435A>T | p.Asp812Val |
