# Supplementary material for: Mutation profiling of 19 candidate genes in acute myeloid leukemia suggests significance of DNMT3A mutations
Source: Oncotarget. 2016 Jun 23;7(34):54825–37. doi: 10.18632/oncotarget.10240 (PMC5342384; doi:10.18632/oncotarget.10240)
Supplement: Supplementary file 4 [file oncotarget-07-54825-s004.docx]

**Supplementary Table S8.** **Primers used for Sanger sequencing**

| ***Gene*** | **exon** | **Forward** | **Reverse** |
| --- | --- | --- | --- |
| *ASXL1* | 11 | AGGCTAAGACTGACCCAGCA | TTCTGATCCTTGGGTTCCTG |
|  | 12-1 | TGCCATGACCCTTAAGCTACT | GAAGGCAGGTCCTCTCTCCT |
|  | 12_2 | CAACTACTGCCGCCTTATCC | TCTTGTCTAGTATCACTTTCCCTCA |
|  | 13_3 | ATTAGGGCTTGGTGGCTCAT | TCACCATTCACCTTGGACAG |
| *CEBPA* | 1_1 | CGCCATGCCGGGAGAACTCT | CTTCTCCTGCTGCCGGCTGT |
|  | 1_2 | GCCGCCTTCAACGACGAGTT | CTTGGCTTCATCCTCCTCGC |
|  | 1_3 | CGGCCGCTGGTGATCAAG | CCCAGGGCGGTCCCACAGC |
|  | 1_4 | CCGCTGGTGATCAAGCAGGA | CCGGTACTCGTTGCTGTTCT |
|  |  | CAAGGCCAAGAAGTCGGTGGACA | CACGGTCTGGGCAAGCCTCGAGAT |
| *DNMT3A* | 2 | GCCTCCAAAGACCACGATAA | CGGCTGTCATCACATAGGG |
|  | 4 | CAGAGCGGTCAATGATCCA | ATCCAGCCAGTAGCCTCCAG |
|  | 9 | TGTAAGCCTCGGCAAACAAG | ACCTGCACTCCAACTTCAG |
|  | 11 | GAAGGGGCTGGAGTTTCCT | TCCCAGGCAACAAACTTACC |
|  | 13 | TTTTTGTGGCTGGTCTGG | CAGAAGCGGTGGACACAGT |
|  | 15 | GCTGAGAGTCTCCTCTGCTCA | CAGGCTCCTAGACCCACACA |
|  | 16 | GCCTGCATCTGACCTGTTGT | CGTTTCCACTTCACACACAAGC |
|  | 17 | GCTCCAAGTAACGGTGCTGT | GGCAAAGGGTGAAGAGAAAG |
|  | 18 | CTTCCTGTCTGCCTGTGTCC | CAAGGAGGAAGCCTATGTGC |
|  | 19 | GCACATAGGCTTCCTCCTTG | TAACCCTGCTTCCTCCCTTT |
|  | 23 | TAAGCAGGCGTCAGAGGAGT | CCATCCTCATGTTCTTGGTG |
| *FLT3*-ITD | 15 | GCAATTTAGGTATGAAAGCCAGC | CTTTCAGCATTTTGACGGCA |
| *FLT3*-TKD | 20 | TCCATCACCGGTACCTCCTA | CCTGAAGCTGCAGAAAAACC |
| *IDH1* | 4 | GAGCTCTATATGCCATCACTGC | TGTGTTGAGATGGACGCCTA |
| *IDH2* | 4 | TCTGGCTGTGTTGTTGCTTG | TGTGGCCTTGTACTGCAGAG |
| *JAK2* | 15 | TGCTGAAAGTAGGAGAAAGTGC | CTGACACCTAGCTGTGATCCTG |
| *KIT* | 17 | TGAACATCATTCAAGGCGTA | TGTTCAGCATACCATGCAAA |
| *KRAS* | 1 | TTAACCTTATGTGTGACATGTTCTAA | AAGAATGGTCCTGCACCAGTAA |
| *NPM1* | 12 | TGTCTATGAAGTGTTGTGGTTCC | TGGCAATAGAACCTGGACAA |
| *NRAS* | 2 | GCCCAAGGACTGTTGAAAAA | TGGGTAAAGATGATCCGACA |
|  | 3 | GGCAGAAATGGGCTTGAATA | CCAAGTCATTCCCAGTAGCAA |
| *PTPN11* | 3 | AAAATCCGACGTGGAAGATG | ACACAGACCGTCATGCATTT |
|  | 7 | AATGCTGATCCAGGCTTTTT | CCCTGAGGAAAGGTACAGAG |
|  | 8 | ACAAGGCAGATCCAACCATC | CCAACTCCTTCATGCACCTC |
|  | 13 | TTGTGCATTAAACAACTTCATCC | CTCCTGCTCAAAAGGAGAGC |

**Supplementary Table S8** ***Continued.***

| **Gene** | **Exon** | **Forward** | **Reverse** |
| --- | --- | --- | --- |
| *RUNX1* | 3 | AGCTGTTTGCAGGGTCCTAA | TATCTTTGGGGACACCCTGA |
|  | 4 | GTTCCAACTGTGCTATGTGACC | CCGAGTTTCTAGGGATTCCA |
|  | 5 | GAAGGGCTGGACAGCATAAA | TCTGAGACATGGTCCCTGAG |
|  | 6 | TGATCTCTTCCCTCCCTCCT | TCTGAGCATCAAGGGGAAAC |
|  | 7 | GAACAAGGGCCACTCATTTC | GAGTCGACTGGAAAGTTCTGC |
|  | 8 | TATCCAGGCGCCTTCACCTA | CCTGACCTACAGCGAGATCC |
| *SETD2* | 1 | GTGTGAGGGTGAGAGGGAGA | CCGCGGAGCTGATACTTACT |
|  | 3_1 | CAACCTATTGGGAGTTCAGG | CTGGAGACGGTTTCTTGGAA |
|  | 3_2 | GGTCACCCAAATTCAGAGGA | GCACTGACCCCTTGTCTTTC |
|  | 8 | ACACACACACACACACACACAC | CAGCCTATTTCCCCATCAGA |
|  | 10 | CCCCAGTCTAGTACTTTCCCAGT | CATTCCCACCACAAAACCAG |
|  | 20 | CCCTCTCTGGACTGTTGAGC | CCAAAAGCAAGGGGAGTACC |
| *TET2* | 3 | CACCCTTGTTCTCCATGACC | CTTGGCTTACCCCGAAGTTA |
|  | 3 | CAGGAGGGGAAAAGTGCTAA | GAGGAACCTGTGGAAGAGG |
|  | 3 | GCAGCAATTTGCAAGCTC | GCAGTGATGCCTCATTACG |
|  | 3 | GGTCGAGACAAGGAGCAAAC | TGTGCTGCCTGTTTATGAGG |
|  | 3 | CCCCAGTGTTGAAACAGCA | CTGCTCCTGTTCTTGAAAGC |
|  | 3 | TGGCCAGACTAAAGTGGAAG | GTTGCTGTGTTTGCTGCTGT |
|  | 3 | TTAAGGTGGAACCTGGATGC | TGCTGCCTAGCTGTCTCTCC |
|  | 6 | GCCCTTATCTGCTGCAAGTG | CGCATGACTGCCAAACAG |
|  | 7 | GTGTGGTTATGCCACAGCTT | TCCTTGGGATCTTGCTTCTG |
|  | 7 | TGGTTGTTCATGGAGCATGT | CAGTTTGGGAAAAACTTTGATT |
|  | 8 | TGGCACAGGCTTGTGTGTAT | GAAGCGCATGTCTTTACAGC |
|  | 10 | TCAACTAGGCCACCAACACA | CTGCATGTGTACCCCAGAAC |
|  | 11 | CATGCTTTCCCACACAGCTA | CCTTTCGGCAAGAGACTTGA |
| *TP53* | 2 | ACCCAGGGTTGGAAGTGTCT | GGGGACTGTAGATGGGTGAA |
|  | 4 | CCTGGTCCTCTGACTGCTCT | GCCAGGCATTGAAGTCTCAT |
|  | 6 | CTGCTCAGATAGCGATGGTG | CTTAACCCCTCCTCCCAGAG |
|  | 7 | CCTGCTTGCCACAGGTCT | CCGGAAATGTGATGAGAG GT |
|  | 8 | AGGCTCCAGAAAGGACAAGG | TAACTGCACCCTTGGTCTCC |
| *WT1* | 7 | TGGTTTTAGGTCTGCACCTG | GCAGTGCTTACTTTCCATCC |
|  | 8 | CCTAACAAGCTCCAGCGAAG | CTGCCAGCAATGAGAAGTGA |
|  | 9 | GGCATGGCAGGAAATGCT | GTATTTCCACCCTCCCCTTC |
